# Light-sheet microscopy with attenuation-compensated propagation-invariant beams

**DOI:** 10.1126/sciadv.aar4817

**Published:** 2018-04-06

**Authors:** Jonathan Nylk, Kaley McCluskey, Miguel A. Preciado, Michael Mazilu, Zhengyi Yang, Frank J. Gunn-Moore, Sanya Aggarwal, Javier A. Tello, David E. K. Ferrier, Kishan Dholakia

**Affiliations:** 1 Scottish Universities Physics Alliance, School of Physics and Astronomy, University of St. Andrews, North Haugh, St. Andrews, Fife KY16 9SS, UK.; 2 School of Biology, University of St. Andrews, North Haugh, St. Andrews, Fife KY16 9ST, UK.; 3 School of Medicine, University of St. Andrews, North Haugh, St. Andrews, Fife KY16 9ST, UK.; 4 Scottish Oceans Institute, Gatty Marine Laboratory, School of Biology, University of St. Andrews, East Sands, St. Andrews, Fife KY16 8LB, UK.

## Abstract

Scattering and absorption limit the penetration of optical fields into tissue. We demonstrate a new approach for increased depth penetration in light-sheet microscopy: attenuation-compensation of the light field. This tailors an exponential intensity increase along the illuminating propagation-invariant field, enabling the redistribution of intensity strategically within a sample to maximize signal and minimize irradiation. A key attribute of this method is that only minimal knowledge of the specimen transmission properties is required. We numerically quantify the imaging capabilities of attenuation-compensated Airy and Bessel light sheets, showing that increased depth penetration is gained without compromising any other beam attributes. This powerful yet straightforward concept, combined with the self-healing properties of the propagation-invariant field, improves the contrast-to-noise ratio of light-sheet microscopy up to eightfold across the entire field of view in thick biological specimens. This improvement can significantly increase the imaging capabilities of light-sheet microscopy techniques using Airy, Bessel, and other propagation-invariant beam types, paving the way for widespread uptake by the biomedical community.

## Teaser

Tailoring beams to overcome attenuation allows light-sheet microscopy to image at greater depth with enhanced contrast.

## INTRODUCTION

The optical analysis of living tissues and organisms has yielded fascinating insights into biological processes. In particular, light-sheet microscopy (LSM) is revolutionizing these studies because of the rapid, high-contrast, wide-field, and minimally phototoxic nature of this imaging method (*1*–*4*). Aberration and absorption in both the excitation and detection pathways are key factors in determining the performance of LSM. If we consider the penetration of light into these specimens, this is ultimately limited by the heterogeneous nature of tissue, making observation within deep tissues problematic. Multiview image fusion methods (*5*, *6*) have become a popular choice for restoring image quality at depth within a specimen but require multiple sequential acquisitions per image, precise rotation, and positioning of the specimen and greatly constrain specimen choice. Separately, a suite of adaptive optical methods has been developed for optical imaging. These typically address the problem by selecting a “guide star” and exploiting the orthogonality of input probe fields to correct for aberrations in a localized area around the guide star (*7*). Although such aberration correction has yielded impressive results in microscopy in general (*7*), it has provided more limited improvements in LSM (*8*–*11*). A major drawback is that the limited region around the corrected point where an improvement is observed is discordant with the wide field of view (FOV) encountered in most LSM systems (*12*). A key step forward with LSM would be to implement an all-optical, single-snapshot acquisition approach to overcome the detrimental effects of tissue absorption and, to a lesser extent, scattering on the incident light field across a wide FOV. This would work in a manner that would reduce the need for, or work in tandem with, adaptive optics. This is what we present here with the use of attenuation-compensated propagation-invariant fields.

Propagation-invariant light fields, most notably Airy (*13*–*15*) and Bessel (*16*–*19*) beams, have gained interest in LSM primarily for their quasi-nondiffracting properties, maintaining a narrow transverse profile over distances much larger than the Rayleigh range of an equivalent Gaussian beam and enabling high-resolution imaging over an increased FOV. The “self-healing” ability of propagation-invariant beams has also been particularly advantageous for imaging. These self-healing beams are able to recompose their transverse profile rapidly on propagation after being partially blocked by an obstacle (*20*–*22*), which has translated to a resistance against aberration in turbid media, with the beam profile degrading at a reduced rate compared to a Gaussian beam (*23*–*25*). Although these fields maintain their profile in the presence of scattering, intensity losses associated with both scattering and absorption still pose a challenge for deep tissue imaging.

The longitudinal intensity envelope of a propagation-invariant field is determined by the method of its generation (*26*), and recent studies have shown that the envelope can be tailored arbitrarily (*26*–*29*). Here, we exploit such control of the longitudinal envelope to counteract attenuation of the illuminating light sheet in optically thick specimens to recover high-contrast images from deeper tissue layers without increased irradiation of tissue layers close to the entry point of the input beams. This attenuation-compensation is achieved by tailoring an exponential increase of intensity along the beam propagation with the exponent matched to the decrease in intensity caused by attenuation.

Using numerical simulations, we characterize the improvement in deep tissue image quality that can be obtained when using attenuation-compensation in LSM with Airy and Bessel beams. We focus on attenuation-compensated Airy LSM to experimentally demonstrate our approach and show deep tissue imaging of *Spirobranchus lamarcki* opercula and sections of mouse brain. In all experiments, we observed a marked increase in signal-to-background ratio (SBR) and contrast-to-noise ratio (CNR), with many indiscernible features being raised noticeably above the noise floor by the use of attenuation-compensation. This, in turn, leads to an extension of the usable, high-contrast FOV by up to 100% (corresponding to an increased depth penetration of 150 μm), even when only partial attenuation-compensation could be achieved.

## RESULTS

### Numerical study of attenuation and attenuation-compensation on image performance

**Fig. 1:**
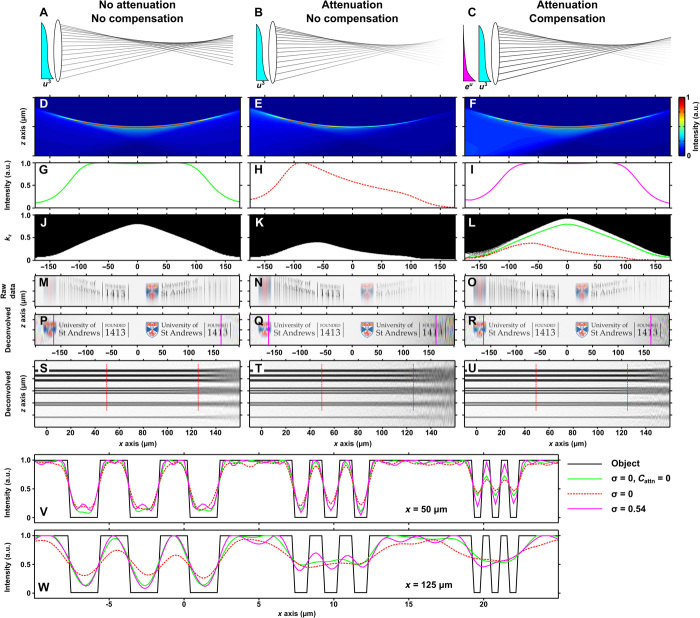
Principle of attenuation-compensation for an Airy light sheet. Ray optics representations of Airy light-sheet formation without (**A**) and with (**B**) attenuation (*C*_attn_ = 65 cm^−1^) and with attenuation-compensation (**C**; σ = 0.54). (**D** to **F**) Wave optical simulations of light-sheet profiles, (**G** to **I**) peak transverse intensity as a function of longitudinal coordinate, and (**J** to **L**) axial MTF thresholded at 5% contrast, respectively, for the light sheets shown in (A) to (C). Green and red lines in (L) match the 5% contour in (J) and (K), respectively. a.u., arbitrary units. (**M** to **R**) Simulated, recorded, and deconvolved images of the University crest and (**S** to **U**) simulated deconvolved images of a 1D resolution target, respectively, for the light sheets shown in (A) to (C). Pink solid lines in (P) to (R) indicate the edge of the FOV from theory. One-dimensional resolution target (P to R) has linewidth/spacing: 2 μm (top) and 1, 0.6, and 0.2 μm (bottom). (**V** and **W**) Intensity profiles through the dashed lines at *x* = 50 and 125 μm in (S) to (U). Simulation parameters were set to mirror experimental parameters (see Materials and Methods).

The intensity of a beam propagating through a linearly attenuating medium (for simplicity, we consider pure absorption; *C*_attn_ ≡ *C*_abs_) will decrease exponentially as given by the Beer-Lambert law, which we show for an Airy light sheet in Fig. 1 (A and B). For propagation-invariant beams, there is a mapping between the transverse coordinate in the pupil plane and the longitudinal coordinate near the focal plane (*26*–*29*). Therefore, by judicious choice of weighted complex amplitudes for different wave vectors of the beam, the intensity profile can be engineered such that the net attenuation is zero (Fig. 1C). The cylindrical pupil function for an attenuation-compensated Airy light sheet is given by

$$P(u)=A_{\sigma }\;\text{exp}(2\pi i\alpha u^{3})\;\text{exp}(-u^{8})\;\text{exp}(\sigma [u-1])H(\sqrt{2}-|u|)$$ (1)

where *u* is the normalized pupil coordinate corresponding to the *z* axis of the microscope, *A*_σ_ is a real scaling factor, α dictates the propagation invariance of the Airy light sheet (*13*), σ dictates the degree of linear attenuation-compensation, *H*(∙) denotes the Heaviside step function, and the light sheet propagates in the positive *x* direction (note S1). Note that Eq. 1 also describes a spherically focused Airy beam, which is then digitally scanned. In Fig. 1C, σ = 0.54 is applied. Similar mappings exist for Bessel beams, and a numerical method to determine the pupil function for an attenuation-compensated Bessel beam is discussed in the Supplementary Materials (note S2).

When attenuation-compensation is applied to an Airy light sheet, the effective attenuation coefficient *C*_attn_′ then becomes

$${C_{\text{attn}}}^{\prime }=C_{\text{attn}}-X$$ (2)

$$X=\frac{{\sigma \text{NA}}^{2}}{3n\alpha \lambda }$$ (3)

where NA is the numerical aperture corresponding to *u* = 1, *n* is the refractive index of the sample, and λ is the vacuum wavelength of the illumination (note S1) (*28*). For Χ = *C*_attn_, the main caustic of the Airy light sheet propagates without any loss.

The selective delivery of additional intensity to greater depths within an attenuating medium allows the Airy light sheet to maintain constant intensity along its main caustic (Fig. 1, D to I). However, with our experimental parameters (see Materials and Methods), we found that for σ > 0.54, the peak intensity was no longer located on the main caustic and so no longer useful for regulating the uniformity of the illumination intensity across the FOV (note S1). Therefore, we defined σ = 0.54 as the maximum permitted attenuation-compensation for this study, allowing complete compensation up to *C*_attn_ = 64 cm^−1^ over the whole FOV of the light sheet as determined from theory (328 μm; Fig. 1, C, F, and I) (*13*). This is well suited for biological microscopy because attenuation coefficients for brain and other tissues are in the range of 50 to 200 cm^−1^ (*30*, *31*), allowing full attenuation-compensation within some tissues and partial attenuation-compensation within others. With different system parameters, it is possible to overcome attenuation greater than *C*_attn_ = 64 cm^−1^, albeit over a shorter FOV. For example, by changing to α = 3, it is possible to apply σ = 0.57, which can compensate for attenuation of *C*_attn_ = 158 cm^−1^ across an FOV of 140 μm or for α = 2 and σ = 0.59; attenuation of *C*_attn_ = 243 cm^−1^ can be counteracted across an FOV of 94 μm.

Airy LSM uses deconvolution of the recorded images based on the point spread function (PSF) of the illuminating light sheet to achieve high axial resolution. Modification of the deconvolution procedure to incorporate the attenuated light-sheet profile (note S3) facilitates correct renormalization of the sample fluorophore distribution but at the cost of amplified noise in regions where the illumination is weak. The axial image quality can be explored through analysis of the modulation transfer function (MTF) of the light-sheet profiles. Figure 1 (J to L) shows the MTF of the Airy light sheets shown in Fig. 1 (A to C) thresholded at 5% to indicate the practical resolution limit of these light sheets in the presence of noise. Figure 1K shows that attenuation results in a marked reduction in the support of high spatial frequencies, and Fig. 1L shows that support for high spatial frequencies is recovered with the use of attenuation-compensation. For complete attenuation-compensation (*C*_attn_′ = 0; Fig. 1L), the bandwidth was found to be higher than for the ideal (nonattenuating) case (Fig. 1J). It is important to note that although this representation of the MTF is indicative of the trend in resolution across the FOV, it does not reveal the absolute resolution.

Figure 1 (M to U) shows simulated raw data (Fig. 1, M to O) and deconvolved images of the University of St. Andrews crest (Fig. 1, P to R) and of a one-dimensional (1D) resolution target (Fig. 1, S to U) for the Airy light sheets shown in Fig. 1 (A to C). Because of the increased intensity and greater support of high spatial frequencies at depth when using attenuation-compensation, the signal is restored without noise amplification. Line profiles along the dashed lines in Fig. 1 (S to U) at *x* = 50 μm and *x* = 125 μm are shown in Fig. 1 (V and W, respectively) (see fig. S3 for more line profiles between *x* = 0 and 150 μm). Whereas the maximum theoretical axial resolution of the Airy light sheet shown in Fig. 1A is 0.77 μm (*13*), Fig. 1V shows that the line triplet at *z* ~ 20 μm (0.6-μm linewidth/spacing) is resolved in all three scenarios at *x* = 50 μm. However, at *x* = 125 μm (Fig. 1W), the line triplet at z ~ 10 μm (1.0-μm linewidth/spacing) is only resolved when attenuation-compensation is used, confirming the recovery of high-frequency details and a marked increase in these details even compared to the noncompensated, nonattenuated case.

Deconvolution requires a priori knowledge of the sample attenuation, and incorrect estimation of *C*_attn_ may result in image artifacts. Estimation of *C*_attn_ to within 10% of the true value was sufficient to minimize the effect of these artifacts, which mainly manifest as a nonuniform intensity envelope across the FOV (note S4). Overestimation of *C*_attn_ additionally resulted in a reduction of high-resolution features, whereas underestimation resulted in amplification of high-frequency noise.

Similarly, the effect of attenuation-compensation on a Bessel beam light sheet can be investigated through analysis of its axial PSF. Because Bessel LSM methods typically do not use deconvolution, analysis of the MTF is not necessary.

**Fig. 2:**
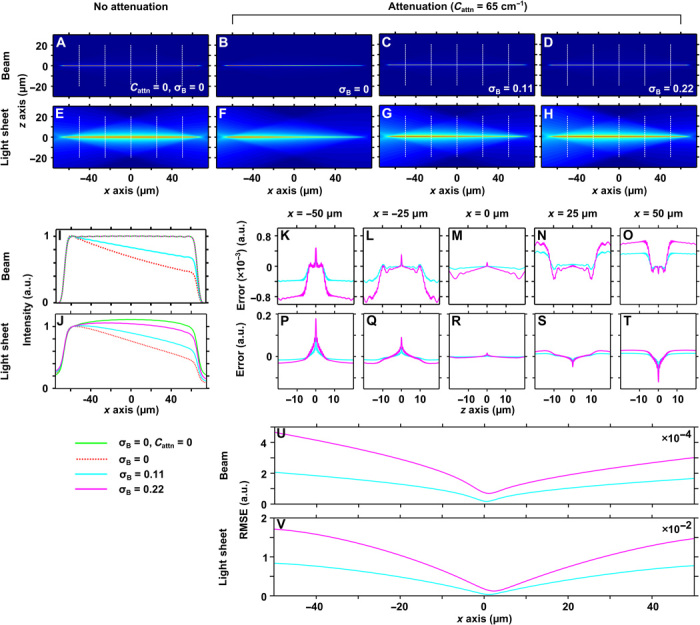
Principle of attenuation-compensation for a Bessel light sheet. Simulated *xz* intensity profiles for a flat-top Bessel beam without (**A**) and with (**B**) attenuation (*C*_attn_ = 65 cm^−1^) and with partial (**C**; σ_B_ = 0.11) and full (**D**; σ_B_ = 0.22) attenuation-compensation. (**E** to **H**) Light-sheet cross sections resulting from digital scanning of the Bessel beams shown in (A) to (D). (**I** and **J**) Peak transverse intensity as a function of longitudinal coordinate for the Bessel beams (A to D) and Bessel light sheets (E to H), respectively. The intensity is normalized to the start of the propagation-invariant region (*x* = −57.5 μm). (**K** to **T**) Comparison of Bessel beam and light-sheet transverse profiles with and without attenuation-compensation at *x* = −50 μm (K and P), −25 μm (L and Q), 0 μm (M and R), 25 μm (N and S), and 50 μm (O and T). (**U** and **V**) RMSE between Bessel beam and light-sheet transverse profiles with and without attenuation-compensation as a function of longitudinal coordinate.

Figure 2 (A and B) shows *xz* cross-sectional profiles for a flat-top Bessel beam (*26*) in nonattenuating and attenuating (*C*_attn_ = 65 cm^−1^) media, respectively. Partially (σ_B_ = 0.11; σ_B_ to differentiate the Bessel compensation parameter from σ, the Airy compensation parameter) and fully (σ_B_ = 0.22) attenuation-compensated Bessel beams are shown in Fig. 2 (C and D, respectively), and the light sheets formed by digital scanning of these beams are shown in Fig. 2 (E to H). Figure 2 (I and J) shows the longitudinal intensity envelopes of the Bessel beams and light sheets shown in Fig. 2 (A to H). For full attenuation-compensation (σ_B_ = 0.22), the intensity envelope of the Bessel beam is completely restored to the ideal (nonattenuated) case (Fig. 2I). However, for the Bessel light sheet with full compensation, the intensity envelope is still attenuated compared to the ideal case (Fig. 2J). Why a fully compensated Bessel beam does not produce a fully compensated light sheet when digitally scanned can be understood as follows: The attenuation-compensation redistributes the field intensity in the 2D transverse plane of the beam, but when the illumination beam is digitally scanned, the generated fluorescence intensity is effectively integrated along one transverse direction, negating any effect of redistribution along this direction. Although this is an important consideration when using digitally scanned Bessel light sheets, Bessel beam LSM modalities are typically based on stepped beams (*16*, *23*, *32*), arrays (*18*, *19*), or multiphoton excitation (*17*), in which cases the fluorescence output depends primarily on the beam profile. We did not observe any deviation of the peak intensity away from the central Bessel core for any combination of σ_B_ and β investigated, allowing compensation of attenuation in excess of *C*_attn_ = 250 cm^−1^ and giving no indication of a practical maximum compensation parameter as was observed for the Airy light sheet (fig. S2). We attribute this to the fact that the energy distribution for an attenuation-compensated Bessel beam happens in the full 2D transverse plane, allowing the additional energy to be dispersed over a larger area than for an attenuation-compensated Airy beam where energy is only dispersed along one dimension in the transverse plane.

To assess the effect of attenuation-compensation on the image quality of Bessel LSM techniques, we compared the *z*-axis cross-sectional profile of the Bessel beams and light sheets with and without compensation for shape similarity. For selected planes [indicated by the dashed lines in Fig. 2 (A, C to E, G, and H)], the variation between the profiles with and without attenuation is shown in Fig. 2 (K to T). The variation (error) was defined as *I*_0_(*z*) – κ*I*_σ_(*z*), where *I*_0_(*z*) and *I*_σ_(*z*) are the cross-sectional intensity profiles without and with compensation, respectively, and κ is a scaling factor to normalize the intensity of each profile, determined by minimizing the error. Figure 2 (K to O) shows that the error at any position does not exceed 0.1% with full compensation, indicating a high fidelity of shape even when attenuation-compensation is applied. Figure 2 (P to T) shows that the instantaneous error can be as much as 20% in the Bessel light sheet. The variation across the FOV, characterized by the root mean squared error (RMSE) in each cross-sectional plane as a function of longitudinal coordinate, is shown in Fig. 2 (U and V). This shows that the average variation across the FOV is <0.05% for an attenuation-compensated Bessel beam and <2% for an attenuation-compensated Bessel light sheet. With or without attenuation-compensation, the full width at half maximum (FWHM) of the Bessel beam core was constant at (0.49 ± 0.04) μm.

### Attenuation-compensated Airy LSM in attenuating phantoms

We focus experimentally on Airy LSM. To experimentally test the performance of attenuation-compensated Airy LSM under strongly attenuating conditions, we developed an attenuation-compensated Airy LSM (see Materials and Methods) and first imaged sub–diffraction-limited (600 nm) fluorescent beads in an absorbing dye solution (*C*_attn_ = 55 ± 1 cm^−1^; see Materials and Methods and notes S5 and S6).

**Fig. 3:**
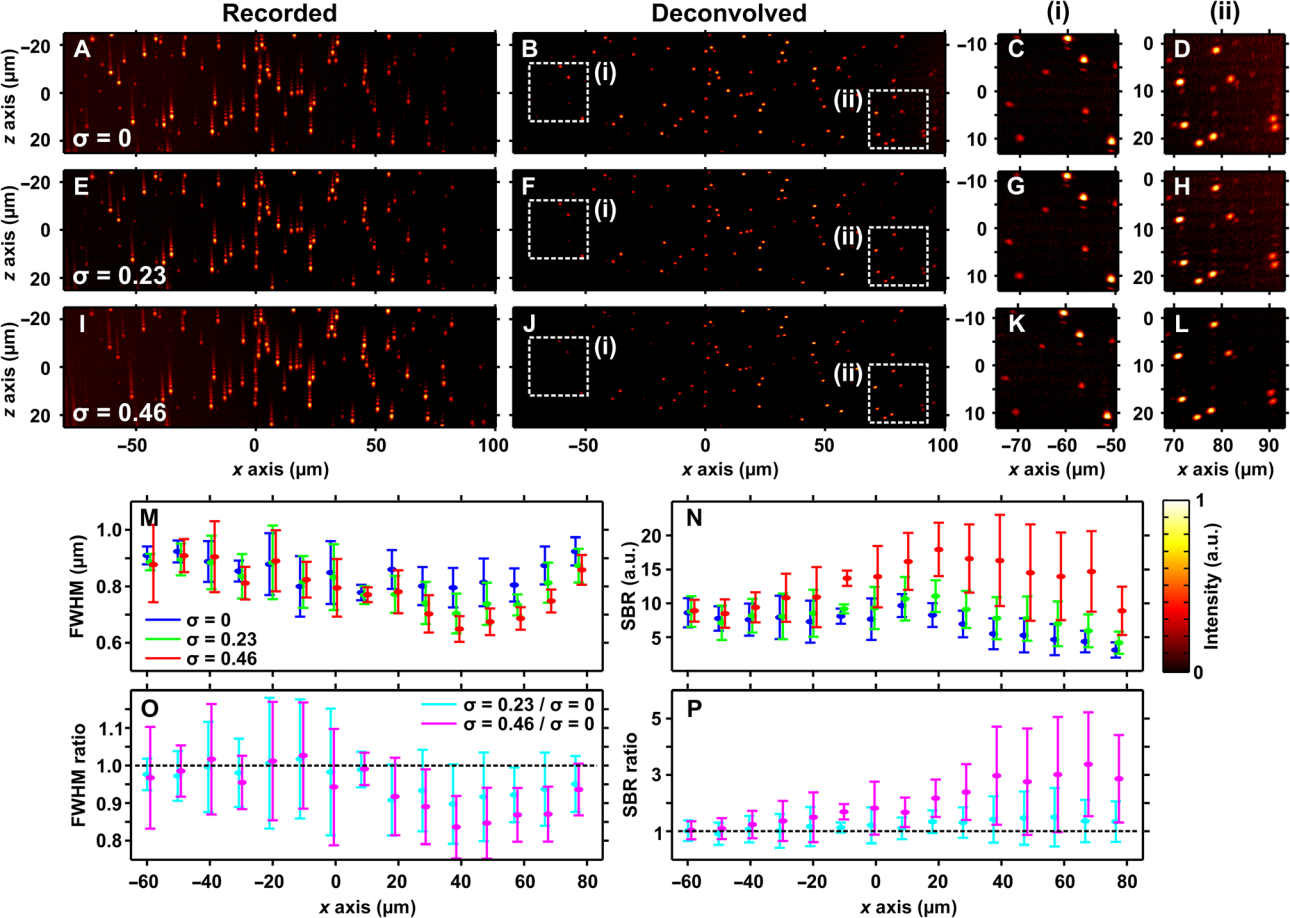
Attenuation-compensated Airy LSM in an attenuating phantom. Maximum intensity projections of recorded data (A, E, and I) and deconvolved images (B, F, and J) of sub–diffraction-limited fluorescent microspheres in an absorbing phantom with *C*_attn_ = 55 ± 1 cm^−1^. (**A** and **B**) No attenuation-compensation (σ = 0), (**E** and **F**) σ = 0.23, and (**I** and **J**) σ = 0.46 (full attenuation-compensation). (**C**, **D**, **G**, **H**, **K**, and **L**) Zoomed-in views of the regions indicated by the dashed boxes (i) and (ii) in (B), (F), and (J). (**M** and **N**) Axial resolution determined by FWHM of fluorescent microspheres and local SBR, respectively, as a function of light-sheet propagation (mean ± SD, 10-μm binning); σ = 0 (blue), σ = 0.23 (green), and σ = 0.46 (red). (**O** and **P**) Ratios of the graphs shown in (M) and (N); σ = 0.23/σ = 0 (cyan) and σ = 0.46/σ = 0 (magenta). Look-up tables of the images shown in (A) to (L) are independently scaled to the data shown.

Figure 3 shows *xz* maximum intensity projections of the recorded data (Fig. 3, A, E, and I) and deconvolved images (Fig. 3, B, F, and J). The sample was imaged with three different levels of compensation: no compensation (σ = 0; *C*_attn_′ = 55 ± 1 cm^−1^; Fig. 3, A to D), partial compensation (σ = 0.23; *C*_attn_′ = 27 ± 1 cm^−1^; Fig. 3, E to H), and full compensation (σ = 0.46; *C*_attn_′ = 0 ± 1 cm^−1^; Fig. 3, I to L). Figure 3 (C, D, G, H, K, and L) shows magnified views of the regions indicated by the dashed boxes (i) and (ii) in Fig. 3 (B, F, and J).

The effect of attenuation-compensation on the raw image intensity is clearly visible. As the strength of the compensation increases, beads deeper into the phantom (positive *x* direction) become visible (Fig. 3, A, E, and I). Because the intensity is renormalized in the deconvolved images, the intensity change is not visible, but an improved SBR (see Materials and Methods) is observed with increasing σ (Fig. 3, B to D, F to H, and J to L).

A spot-finding algorithm (see Materials and Methods) was used to identify and measure individual beads in the data from Fig. 3 (B, F, and J). Figure 3 (M and N) shows the axial FWHM and the local SBR for each compensation level, and Fig. 3 (O and P) shows ratios of the data relative to the case of σ = 0. Results of individual beads were aggregated in 10-μm intervals along the *x* axis, and the mean and SD within each interval are shown. The lateral resolution for all compensation levels was 1.2 ± 0.2 μm with no trend across the FOV. This is larger than expected for diffraction-limited performance but consistent with a small amount of spherical aberration, which was expected because of the walls of the capillary tube housing the sample (*13*). The large variance of SBR observed (Fig. 3, N and P) is expected because of the variance in bead brightness.

We additionally characterized the 3D imaging performance of attenuation-compensated Airy LSM in a sample where attenuation is primarily caused by scattering rather than absorption. For this, 600-nm red fluorescent beads were suspended in a 1% agarose gel cast around a 300-μm-thick section of mouse brain tissue (not subjected to previous adenoassociated virus vector infusion). By orienting the sample and varying the distance that the illumination beam passes through the tissue before encountering the beads, the distance can be easily adjusted from 0 (above the tissue and down to the top edge) to √2 × 300 μm (through the bottom edge of the tissue). This allowed us to evaluate the performance of the attenuation-compensated Airy LSM at increasing tissue depth. We isolated single beads both in recorded data and in deconvolved images, illuminated through brain tissue thicknesses of up to 60 μm and compared between Airy LSM with (σ = 0.54) and without (σ = 0) attenuation-compensation (fig. S6). Whereas a steady reduction in the quality of the recorded PSF and its deconvolved image was observed with increasing tissue thickness, the 3D shape of the PSF is effectively unchanged whether attenuation-compensation is used or not, indicating that the 3D imaging capability of Airy LSM is not adversely affected by the use of attenuation-compensation.

### Attenuation-compensated Airy LSM in thick biological specimens

Next, we tested the performance of attenuation-compensation in a range of thick biological specimens where attenuation is the result of a combination of absorption and scattering. In all specimens shown, the attenuation exceeds the maximum that can be fully compensated across the FOV, given the system parameters of our microscope (see Materials and Methods).

First, we imaged the cellular arrangement within the operculum of *S*. *lamarcki*. The polychaete *S*. *lamarcki* (formerly *Pomatoceros lamarckii*) is a serpulid tubeworm that is found widely in temperate marine habitats, often in large numbers in rocky intertidal zones or biofouling man-made marine infrastructure. In recent years, the animal has been used to better understand aspects of animal genome evolution and evolutionary developmental biology, including appendage regeneration (*33*). The operculum is an appendage on the head of *S*. *lamarcki* that is used as a protective plug for the habitation tube when the animal is threatened and is particularly accessible and amenable as a study system for regeneration. Improved methods of imaging this appendage are particularly valuable in this context.

**Fig. 4:**
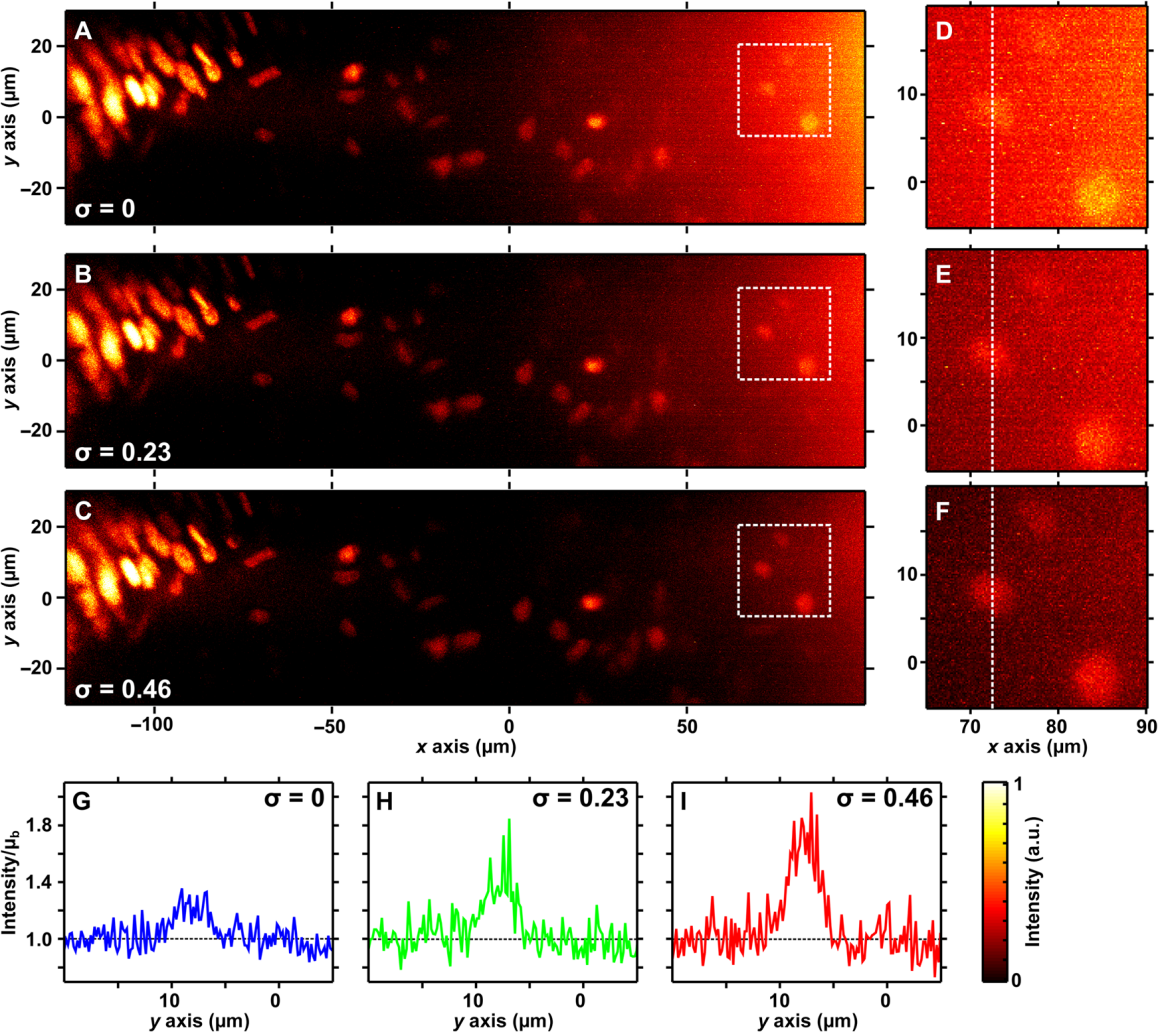
Attenuation-compensated Airy LSM in *S*. *lamarcki* opercula. Maximum intensity projections of deconvolved Airy LSM images of nuclei stained with propidium iodide (PI) in the operculum of *S*. *lamarcki* (attenuation estimated at 85 cm^−1^) with (**A**) no attenuation-compensation, (**B**) σ = 0.23, and (**C**) σ = 0.46. (**D** to **F**) Expanded views of the region indicated by the dashed box in (A) to (C). (**G** to **I**) Intensity profiles along the dashed line shown in (D) to (F). Line intensity profiles are shown relative to the noise floor, given by the local mean background μ_b_.

Figure 4 compares images of the operculum at different levels of attenuation-compensation. In Fig. 4A, no compensation is applied (*C*_attn_′ = 85 cm^−1^). Compensation with σ = 0.23 (*C*_attn_′ = 57 cm^−1^) and σ = 0.46 (*C*_attn_′ = 30 cm^−1^) is shown in Fig. 4 (B and C). Although full compensation cannot be achieved in this sample, increasing σ steadily improves the SBR at depth in the tissue. Figure 4 (D to F) shows expanded views of a region ~200 μm deep into the tissue, indicated by the dashed box in Fig. 4 (A to C). Without compensation, these deep nuclei are only marginally above the noise floor, but for σ = 0.46, the SBR is increased between 20 and 45% and the CNR is increased between 20 and 140% (see Materials and Methods and fig. S**7**), raising features sufficiently above the noise floor for easy identification. Figure 4 (G to I) shows the intensity profile along the dashed line in Fig. 4 (D to F), normalized to the noise floor, again highlighting the marked improvement in SBR and CNR with attenuation-compensation.

**Fig. 5:**
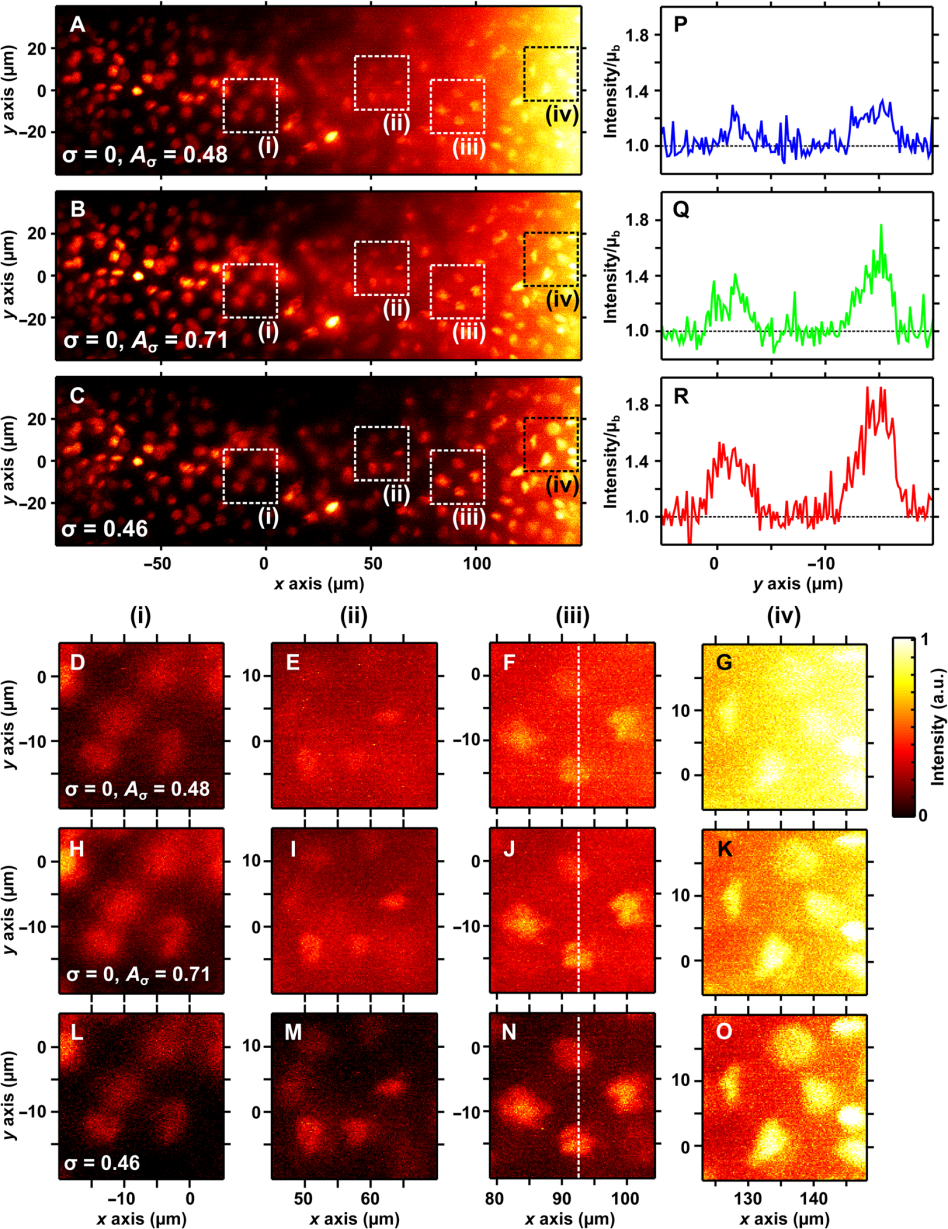
Attenuation-compensated Airy LSM in *S. lamarcki* opercula. Maximum intensity projections of deconvolved Airy LSM images of nuclei stained with PI in the operculum of *S. lamarcki* (attenuation estimated at 75 cm^−1^) with (**A**) no attenuation-compensation, (**B**) no attenuation-compensation but the same total power as a compensated light sheet (σ = 0.46), and (**C**) σ = 0.46. (**D** to **G**) Zoomed-in views of the region indicated by dashed boxes (i) to (iv) in (A). (**H** to **O**) Same regions from (B) and (C). Intensity profiles along the dashed line in (F), (J), and (N) are shown in (**P**) to (**R**), respectively. Line intensity profiles are shown relative to the noise floor, given by the local mean background μ_b_.

We also compared the effect of attenuation-compensation to the “naïve” case in which more intensity is delivered deeper into the tissue simply by increasing the overall illumination power at the back aperture of the objective to match that of the attenuation-compensated beam. Because high illumination intensity is linked to increased photobleaching and phototoxicity, lower illumination intensities are preferable. In Fig. 5A, a different region of the *S*. *lamarcki* operculum is shown without attenuation-compensation (*C*_attn_ = 75 cm^−1^). In Fig. 5B, no attenuation-compensation is used, but the illumination power has been increased to match that of an attenuation-compensated light sheet (σ = 0.46; see Fig. 5C and table S1). Expanded views of the regions indicated by dashed boxes (i) to (iv) in Fig. 5 (A to C) are shown in Fig. 5 (D to O). Intensity profiles along the dashed line in Fig. 5 (F, J, and N), normalized to the noise floor, are shown in Fig. 5 (P to R, respectively). In regions (ii) to (iv), the use of increased power without attenuation-compensation increased the SBR by 0 to 20% and the CNR by 0 to 150%, whereas the use of attenuation-compensation increased the SBR by 20 to 40% and the CNR by 50 to 650% (fig. S8). Without attenuation-compensation, the SBR is only above 1.5 and CNR above 2 in region (i), whereas with attenuation-compensation, this is achieved even in region (iv), effectively increasing the useable, high-contrast FOV by 150 μm (~100%).

Although increasing the power of the noncompensated beam (Fig. 5, B, H to K, and Q) does improve the SBR and CNR at depth, it does so at the cost of increased irradiation of superficial tissues (fig. S9), whereas the use of attenuation-compensation (Fig. 5, C, L to O, and R) increases the SBR and CNR at depth more, greatly assisting feature recognition, and does so with relatively uniform irradiation across the FOV (fig. S9). Assuming that the laser power can be adjusted until the desired signal at a specific depth can be achieved, the main concern then should be to minimize irradiation throughout the rest of the specimen to limit phototoxicity. Attenuation-compensation maintains uniform (or as close to that, which can be achieved in the case of partial compensation) intensity across the specimen.

Finally, we imaged fluorescently labeled kisspeptin neurons in the hypothalamic arcuate nucleus of mouse brain sections. Kisspeptins are a family of brain hormones that are necessary for pubertal development and the maintenance of fertility in humans and mice (*34*, *35*). Sex steroids control reproductive physiology through direct actions on kisspeptin neurons in the hypothalamus. One population of kisspeptin neurons located in the arcuate nucleus displays acute structural plasticity to circulating sex steroids; low sex steroid levels lead to cell hypertrophy, whereas chronically high steroid levels lead to reduced cell size and decreased dendritic spine density (*36*). Studies have recently linked dysregulated kisspeptin neuronal signaling to increased accumulation of visceral fat, glucose intolerance, and menopausal hot flushes, indicating that appropriate kisspeptin brain functions are important for preventing diseases not only associated with the reproductive system but also diabetes, energy imbalance, and thermoregulatory disorders. Improved methods for imaging the interaction of kisspeptin neurons throughout the brain will be beneficial for neuroendocrine research in particular and for neuroscience in general.

We switched from a 2+1D Airy digital scanned light-sheet microscopy (DSLM) system to a 1+1D Airy selective plane illumination microscopy (SPIM) system (see Materials and Methods) to capture the long-range processes of kisspeptin neurons. The systems are identical in operation and performance except for the *y*-axis extent of the light sheet, which is at least four times larger in our SPIM system than in our DSLM system.

**Fig. 6:**
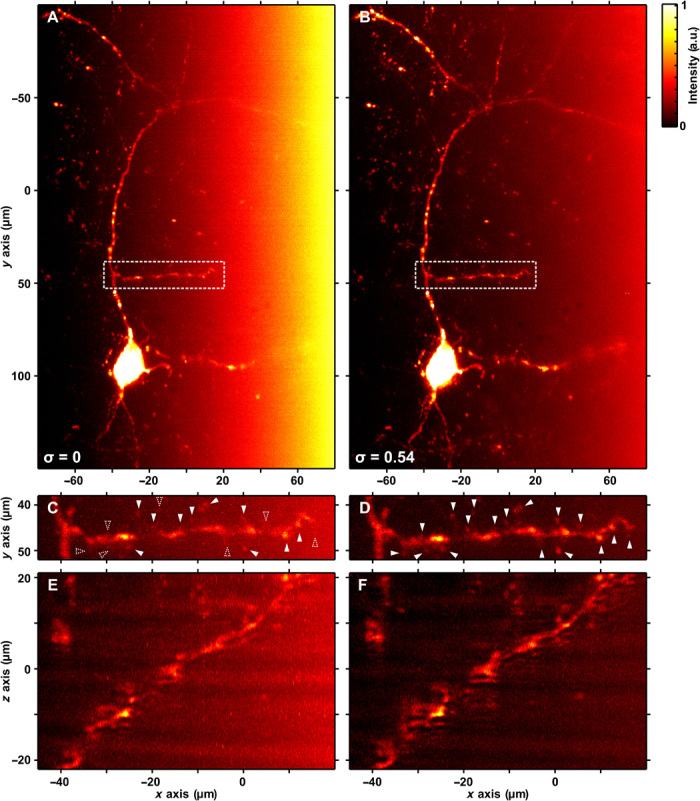
Attenuation-compensated Airy LSM in mouse brain section. Maximum intensity projections of deconvolved Airy LSM images of kisspeptin neurons expressing mCherry in the hypothalamic arcuate nucleus of a mouse brain (attenuation estimated at 100 cm^−1^) with (**A**) no attenuation-compensation and (**B**) σ = 0.54. (**C** and **D**) Expanded views of the region indicated by the dashed box in (A) and (B). (**E** and **F**) Orthogonal projections of the regions shown in (C) and (D). Filled arrowheads indicate the positions of dendritic spines in (C) and (D). Dashed arrowheads in (C) indicate the position of dendritic spines not identified without attenuation-compensation.

Figure 6 shows an isolated kisspeptin soma and its processes. The attenuation of this tissue section has been estimated at *C*_attn_ = 100 cm^−1^. In Fig. 6A, no compensation has been applied. Attenuation-compensation (σ = 0.54, *C*_attn_′ = 35 cm^−1^) is applied in Fig. 6B. Figure 6 (C and D) shows expanded views of the region indicated by the dashed box in Fig. 6 (A and B), and Fig. 6 (E and F) shows orthogonal projections of this region.

The increased contrast in the attenuation-compensated image allows a more accurate count of dendritic spines to be made. Filled arrowheads in Fig. 6 (C and D) indicate the positions of dendritic spines. Dashed arrowheads in Fig. 6C indicate the position of dendritic spines not identified without attenuation-compensation. We manually identified 17 dendritic spines in Fig. 6D but only 10 without attenuation-compensation (Fig. 6C).

## DISCUSSION

We have taken advantage of attenuation-compensation, an intrinsic property of propagation-invariant beams, and applied this both to maximize signal content within turbid specimens and, at the same time, to minimize specimen irradiation. Through numerical simulations, we have shown that attenuation-compensation redistributes the intensity of Airy and Bessel light sheets, thereby enhancing image quality at depth in specimens, without any deleterious side effects.

Attenuation-compensated Airy LSM improved feature definition at depths of 50 to 200 μm in *S*. *lamarcki* opercula and sections of mouse brain tissue, which we quantified using the local SBR and CNR. This effectively increased the high-contrast FOV that could be achieved by ~100%. In mouse brain tissue, attenuation-compensation enabled the clear identification of seven additional dendritic spines (an additional 70%) that could not be distinguished from background without our approach (Fig. 6, C and D). Both the specimens used in this study had measured attenuation coefficients greater than the maximum we could compensate for. However, in both cases, even partial attenuation-compensation resulted in marked improvements in image quality.

Although LSM, in general, minimizes photobleaching and phototoxicity compared to other imaging modalities (*37*, *38*), the importance of maintaining low peak intensity throughout the FOV has been highlighted, and relatively small differences (a factor of 2 to 4) in intensity have shown a marked difference in photobleaching rate (*13*, *19*). The variation of peak illumination intensity across the FOV is minimized when full compensation can be achieved. If the attenuation is not completely matched, then undercompensation or overcompensation can occur, and the intensity variance across the FOV will increase in superficial or deeper tissues. We have seen that even when undercompensated, the intensity variance across the FOV is reduced; therefore, the potential for photodamage is also reduced. We also postulate that overcompensation may be useful in some scenarios. For example, when a region of interest (ROI) is identified within a specimen, the use of an overcompensated light sheet would allow the intensity in superficial areas to be reduced, thereby reducing phototoxicity, without sacrificing signal from the ROI. Therefore, attenuation-compensation in LSM may be an innovative route to low phototoxicity in deep tissue imaging.

Although our system uses a dynamically reconfigurable spatial light modulator (SLM), allowing the strength of attenuation-compensation to be varied and tuned, all biological specimens exhibited attenuation in excess of the maximum compensation that can be applied in this way. Practically, in most studies, it may be possible to use a fixed optical element with no need to adjust σ, yielding a light sheet with a fixed compensation profile. Use of tailored linear neutral density filters may be suitable for low-cost implementation of attenuation-compensation in an Airy LSM system. Because of the complex 2D amplitude and phase profile required for producing attenuation-compensated Bessel beams (note S2), low-cost implementations for Bessel LSM techniques may not be as readily achievable.

The proposed approach for attenuation-compensation of Airy and Bessel LSM in the single-photon excitation regime counteracts the attenuation of the illumination, which is composed of both absorption and scattering. Even assuming a constant attenuation coefficient for a specimen, the specimen geometry can have a marked effect on the perceived attenuation profile across the FOV (see note S6 and fig. S5). In addition, the heterogeneity of scattering will cause small-scale deviations away from a general, homogeneous attenuation profile. In our analysis, we neglect these small-scale deviations and apply attenuation-compensation to counteract an exponential intensity decay. The shape of the amplitude profile that gives attenuation-compensation can be arbitrary and can therefore compensate for any attenuation profile. Full characterization of the specimen transmission properties and its attenuation profile could yield greater improvements in image quality, although the use of a simplified model as presented here can still give impressive results.

Attenuation-compensation may be even more crucial in the multiphoton excitation regime, because absorption losses dominate at the longer illumination wavelengths required. Because of the nonlinear intensity dependence of two-photon excitation fluorescence signals, we anticipate the usable FOV of these techniques to reduce to less than half in the presence of strong attenuation (note S7). This is the subject of a future study.

Overall, this is a powerful, easy-to-implement approach that needs only a simplified model of the transmission properties of the specimen, is not restricted to any “point”, readily elevates the SBR, CNR, and information content from wide-field LSM using propagation invariant beams, and is fully compatible with, and complimentary to, aberration correction methods. We anticipate that this will find significant uptake in LSM, particularly techniques exploiting Airy, Bessel, and potentially lattice modalities. We also note that this approach may be used for studies of propagation-invariant beams in optical manipulation, optical coherence tomography, and other forms of imaging.

## MATERIALS AND METHODS

### Numerical simulations

Numerical simulations, where possible, matched experimental parameters. Light-sheet profiles were generated either by Fourier beam propagation of a 2D (spherical) pupil function and then integrated along the *y* axis or directly by Fourier beam propagation of a 1D (cylindrical) pupil function. Attenuation was modeled by propagation through a linearly absorbing medium where appropriate.

#### Airy LSM

Airy light sheets were simulated from the 1D pupil function given by Eq. 1 with the following parameters: λ = 532 nm, α = 7, NA = 0.42, and *n* = 1.33. This yielded an Airy light sheet with an FOV of 328 μm and matched experimental parameters. Imaging was simulated as a convolution, along the *z* axis, of the sample fluorophore distribution with the light-sheet profile.

#### Bessel LSM

Flat-top Bessel beams were simulated from the 2D pupil function generated by the numerical method given in note S2 (*26*), with the following parameters: λ = 532 nm, β = 0.05, NA = 0.42, and *n* = 1.33, where β dictates the propagation-invariant length of the Bessel beam and is given as the ratio of the thickness of an annular ring to the radius of its outer edge, which is used to form the Bessel beam in the Fourier plane (*13*). This yielded a Bessel light sheet with an FOV of 115 μm.

### Attenuation-compensated Airy light-sheet microscope

The Airy light-sheet microscope is similar to that described in the study of Vettenburg *et al*. (*13*) and can operate in either SPIM or DLSM modality. The optical setup is shown in fig. S13. In brief, we generated the attenuation-compensated Airy light sheet via an SLM (Hamamatsu LCOS X10468-04). The excitation laser (Laser Quantum Finesse, 5 W, 532 nm) was expanded to overfill the active area of the SLM, programmed to display the appropriate phase mask (Eq. 1; see note S1), and modulated in the first-order of a blazed diffraction grating. The SLM was imaged onto an acousto-optic deflector (AOD; Neos AOBD 45035-3) and then onto the back aperture of the illumination objective lens (Nikon CFI Apo 40×/0.80 DIC; working distance, 3.5 mm; water immersion). Pinholes were placed in each relay telescope at the Fourier plane to select only the first-order beams from the SLM and AOD. The SLM was used to holographically control the NA of the illumination. For all experiments, NA = 0.42 was set to correspond to *u* = 1 (note S1) and α = 7 was used, which yielded a maximum FOV of 328 μm (*13*).

The identical illumination and detection objective lenses were mounted in a “dual-inverted” geometry. Collected fluorescence was imaged onto a scientific complementary metal-oxide semiconductor camera (Hamamatsu Orca-Flash4.0 v2) via a tube lens (Thorlabs, TTL200). A pinhole at the back aperture of the detection objective lens restricted the NA to 0.4. To retain the *z* axis as the standard axial coordinate of the microscope, we defined the *x* axis as the propagation direction of the illuminating light sheet, and *x* = 0 is the position where the focus of a Gaussian light sheet would be located. Data acquisition software was written in-house in LabVIEW, and data processing and deconvolution software was written in-house in MATLAB.

#### 2+1D Airy DSLM

For DSLM operation, the SLM generated the phase profile for a 2+1D Airy beam, the beam was spherically focused into the sample with pupil function described by eq. S7, and the light sheet was formed by rapid modulation of the first-order deflection angle from the AOD. Because of the limited modulation range, the *y*-axis extent of the light sheet was limited to ~100 μm.

#### 1+1D Airy SPIM

For SPIM operation, the SLM generated the phase profile for a 1+1D Airy beam, and a cylindrical lens (Thorlabs, ACY254-050-A) was inserted before the illumination objective to form a line focus at the back aperture. The light sheet was then directly formed by cylindrical focusing with pupil function described by eq. S6. The *y*-axis extent of the light sheet exceeded the FOV of the camera (356 μm).

### Fluorescent attenuating phantoms

Strongly attenuating (absorbing) phantoms containing resolution markers were made by adding red fluorescent beads (600 nm in diameter; Duke Scientific, R600) to 6.92 mM Neutral Red dye (ε = 15.9 ± 0.2 mM^−1^ cm^−1^). The suspension was mixed with an equal volume of 1% low–melting point agarose and injected into a square-profile borosilicate capillary (Hawksley, Vitrotube 8250-100), which was sealed at the ends with putty (Hawksley, Crystaseal). The final concentration of Neutral Red in the phantom was 3.46 mM (*C*_abs_ = 55 ± 1 cm^−1^).

### Image analysis

#### Spot-finding algorithm

Our spot-finding algorithm was implemented in MATLAB. We raster-scanned through a deconvolved data cube and identified any pixel whose intensity was above a preset threshold value. We checked that this pixel was the maximum within a 19 × 19 pixel (3 × 3 μm^2^) square, recentered on the true maximum if necessary, and fitted a 3D Gaussian function. Spots with FWHM beyond the range of 0.5 to 3 μm were deemed to be noise or not a single isolated bead and were discarded from the analysis.

To increase the speed of the algorithm, we performed the raster scan on an *xy* maximum intensity projection, only scanning along the third dimension when a local maximum was detected. We also logged the (*x*, *y*, *z*) pixel coordinates of the center of each spot and deleted any duplicates from the final list.

#### Signal-to-background ratio

The local SBR is given by

$$\text{SBR}\;=\mu_{s}/\mu_{b}$$ (4)

where μ_s_ is the mean pixel value within a given ROI identified as a structure of interest or “signal” and μ_b_ is the mean pixel value within an ROI of equal size identified as containing no structures of interest or “background.” Typically, the ROIs chosen were 3 × 3 μm^2^ squares.

#### Contrast-to-noise ratio

The local CNR is given by

$$\text{CNR}\;=\frac{\mu_{s}-\mu_{b}}{\sqrt{\sigma_{s}^{2}+\sigma_{b}^{2}}}$$ (5)

where μ_s_ and μ_b_ are the mean pixel values in signal and background ROIs as described above and σ_s_ and σ_b_ are the SD of the pixel values in the signal and background ROIs, respectively (*39*). Typically, the ROIs chosen were 3 × 3 μm^2^ squares.

### *S. lamarcki* opercula

#### Animals

Adults of polychaete *S*. *lamarcki* (formerly *P*. *lamarckii*) were collected at East Sands, St. Andrews, and maintained in the circulating seawater aquarium system of the Scottish Oceans Institute, Gatty Marine Laboratory, at close to ambient seawater temperature.

#### Tissue collection

Adults were removed from their calcareous habitation tube by crushing the posterior end of the tube until the opening was wide enough for the animal to pass through and then by using a blunt probe to push the animal out of the remaining tube from the anterior end. Animals were then rinsed in fresh filtered seawater, and opercula were removed with a scalpel, cutting at the Easy Break Point (*33*). Isolated opercula were rinsed in filtered seawater, and then as much of the water was removed as possible before fixing in 4% paraformaldehyde (PFA) in 1× phosphate-buffered saline (1× PBS). The opercula were gently rotated in the fixative for 5 min before replacing with fresh fixative solution and fixing overnight at 4°C. The fixed opercula were then given two washes in 70% ethanol, with gentle rotation at room temperature (RT), for 5 min each wash. This was repeated with absolute ethanol for two further 5-min washes. The opercula were then kept in 70% ethanol until used for PI staining.

#### Fluorescent labeling

For labeling with PI, half of the 70% ethanol was replaced with PBT (1× PBS + 0.1% Triton X-100) and gently rotated for 5 min at RT. This step was repeated, and then all the solution was replaced with PBT and rotated gently for 5 min at RT. The opercula were then washed in fresh PBT with gentle rotation for at least 1 hour. Two 2× saline sodium citrate (2× SSC) washes of 5 min at RT with gentle rotation followed before treatment with RNAseA (100 μg/ml) in 2× SSC for 20 min at 37°C. The RNAseA was removed by three 5-min washes with 2× SSC at RT with gentle rotation, and the opercula were stained with PI using a 1:1000 dilution of a stock (1 mg/ml) diluted in 2× SSC. The staining was carried out for 30 min in the dark at RT, and excess PI was then removed by three 5-min washes in 2× SSC at RT with gentle rotation in the dark. Samples were kept in 2× SSC in the dark at 4°C until imaged.

#### Imaging

For imaging, samples were placed on a microscope slide, embedded in 1% low–melting point agarose, and immersed in 1× PBS on the microscope sample stage.

### Mouse brain sections

#### Animals

All rodent experiments were reviewed and approved by the University of St. Andrews Animal Ethics and Welfare Committee under J.A.T.’s Home Office Project License 70/7924. Adult female heterozygous *Kiss1*^tm1.1(cre/EGFP)Stei^/J mice (10 to 12 weeks; 20 to 25 g) were used for this study. The mice were housed in a temperature- and humidity-controlled environment under regular light-dark cycles (12 hours light, 12 hours dark) with food and water available ad libitum.

#### In vivo AAV infusion

Mice were anesthetized with isofluorane and placed in a stereotaxic apparatus. To selectively express mCherry (a monomeric red fluorescent protein) in arcuate *Kiss1*^+^ neurons, we used a Cre recombinase (Cre)–dependent adenoassociated virus vector (*40*) [AAV; AAV1/2-Ef1a-DIO-mCherry-wPRE as described previously (*25*)]. Viral particles were injected bilaterally into the hypothalamic arcuate nucleus (coordinates: AP, 1.7; ML, ±0.3; DV, −5.9) using a pulled glass pipette at a volume of 400 nl per side, at a rate of 100 nl/min using pressure injection. After surgery, the mice were returned to their cages for 3 weeks to allow for AAV-mediated mCherry expression.

#### Perfusion and tissue sectioning

After 3 weeks, the mice were administered with an overdose of sodium pentobarbital (100 mg/kg) and transcardially perfused with 0.1 M PBS (pH 7.4) followed by 4% PFA in PBS (pH 7.4). Brains were removed from the skull and postfixed overnight in 4% PFA in PBS. The brains were sectioned using a Compresstome vibratome (Precisionary Instruments VF-300) at a thickness of 300 μm.

#### Imaging

For imaging, tissue sections were placed on a microscope slide, embedded in 1% low–melting point agarose, and immersed in 1× PBS on the microscope sample stage.

## Supplementary Material

http://advances.sciencemag.org/cgi/content/full/4/4/eaar4817/DC1

## SUPPLEMENTARY MATERIALS

Supplementary material for this article is available at http://advances.sciencemag.org/cgi/content/full/4/4/eaar4817/DC1 note S1. Attenuation-compensation of an Airy beam light sheet note S2. Attenuation-compensation of a Bessel beam light sheet note S3. Modification of deconvolution protocol incorporating attenuation and attenuation-compensation note S4. Effect of incorrect attenuation estimation on deconvolution note S5. Determination of specimen attenuation note S6. Sample-based geometric effects on attenuation note S7. Attenuation-compensation of multiphoton excitation Airy and Bessel light sheets fig. S1. Pupil functions of attenuation-compensated Airy and Bessel beams. fig. S2. Look-up tables of achievable attenuation-compensation for Airy and Bessel beam parameters. fig. S3. Line profiles through simulated images of a 1D resolution target shown in Fig. 1 (main text). fig. S4. Simulated images: Effect on deconvolution from error in estimation of attenuation. fig. S5. Sample induced geometric effects on attenuation profile across the FOV. fig. S6. Attenuation-compensated LSM in a scattering sample. fig. S7. Local SBR and CNR measured for data shown in Fig. 4 (main text). fig. S8. Local SBR and CNR measured for data shown in Fig. 5 (main text). fig. S9. Light-sheet intensity profiles for the data shown in Fig. 5 (main text). fig. S10. Effect of attenuation and attenuation-compensation on two-photon excitation SPIM and DLSM Airy light sheet. fig. S11. Effect of attenuation and attenuation-compensation on two-photon excitation Bessel beam. fig. S12. Effect of attenuation and attenuation-compensation on two-photon excitation DSLM Bessel light sheet. fig. S13. Schematic of attenuation-compensated Airy light-sheet microscope. table S1. Experimental parameters for all data shown in main text. table S2. FOV of two-photon excitation SPIM and DLSM Airy light sheet with attenuation and attenuation-compensation. table S3. FOV of two-photon excitation Bessel beam and DLSM Bessel light sheet with attenuation and attenuation-compensation.
