# Supplementary material for: Light-sheet microscopy with attenuation-compensated propagation-invariant beams
Source: Sci Adv. 2018 Apr 6;4(4):eaar4817. doi: 10.1126/sciadv.aar4817 (PMC5938225; doi:10.1126/sciadv.aar4817)
Supplement: http://advances.sciencemag.org/cgi/content/full/4/4/eaar4817/DC1 [file supp_4_4_eaar4817__index.html]

Science Advances | Science Advances

## Supplementary Materials

**This PDF file includes:**

- note S1. Attenuation-compensation of an Airy beam light sheet
- note S2. Attenuation-compensation of a Bessel beam light sheet
- note S3. Modification of deconvolution protocol incorporating attenuation and attenuation-compensation
- note S4. Effect of incorrect attenuation estimation on deconvolution
- note S5. Determination of specimen attenuation
- note S6. Sample-based geometric effects on attenuation
- note S7. Attenuation-compensation of multiphoton excitation Airy and Bessel light sheets
- fig. S1. Pupil functions of attenuation-compensated Airy and Bessel beams.
- fig. S2. Look-up tables of achievable attenuation-compensation for Airy and Bessel beam parameters.
- fig. S3. Line profiles through simulated images of a 1D resolution target shown in Fig. 1 (main text).
- fig. S4. Simulated images: Effect on deconvolution from error in estimation of attenuation.
- fig. S5. Sample induced geometric effects on attenuation profile across the FOV.
- fig. S6. Attenuation-compensated LSM in a scattering sample.
- fig. S7. Local SBR and CNR measured for data shown in Fig. 4 (main text).
- fig. S8. Local SBR and CNR measured for data shown in Fig. 5 (main text).
- fig. S9. Light-sheet intensity profiles for the data shown in Fig. 5 (main text).
- fig. S10. Effect of attenuation and attenuation-compensation on two-photon excitation SPIM and DLSM Airy light sheet.
- fig. S11. Effect of attenuation and attenuation-compensation on two-photon excitation Bessel beam.
- fig. S12. Effect of attenuation and attenuation-compensation on two-photon excitation DSLM Bessel light sheet.
- fig. S13. Schematic of attenuation-compensated Airy light-sheet microscope.
- table S1. Experimental parameters for all data shown in main text.
- table S2. FOV of two-photon excitation SPIM and DLSM Airy light sheet with attenuation and attenuation-compensation.
- table S3. FOV of two-photon excitation Bessel beam and DLSM Bessel light sheet with attenuation and attenuation-compensation.

Download PDF

**Files in this Data Supplement:**

- Adobe PDF - aar4817\_SM.pdf
