# Supplementary material for: Light-sheet microscopy with attenuation-compensated propagation-invariant beams
Source: Sci Adv. 2018 Apr 6;4(4):eaar4817. doi: 10.1126/sciadv.aar4817 (PMC5938225; doi:10.1126/sciadv.aar4817)
Supplement: http://advances.sciencemag.org/cgi/content/full/4/4/eaar4817/DC1 [file aar4817_SM.pdf]

Science Advances

AAAS

advances.sciencemag.org/cgi/content/full/4/4/eaar4817/DC1

Supplementary Materials for

Light-sheet microscopy with attenuation-compensated propagation-invariant beams

Jonathan Nylk, Kaley McCluskey, Miguel A. Preciado, Michael Mazilu, Zhengyi Yang, Frank J. Gunn-Moore, Sanya Aggarwal, Javier A. Tello, David E. K. Ferrier, Kishan Dholakia

Published 6 April 2018, *Sci. Adv.* **4**, eaar4817 (2018)
DOI: 10.1126/sciadv.aar4817

This PDF file includes:

- note S1. Attenuation-compensation of an Airy beam light sheet
- note S2. Attenuation-compensation of a Bessel beam light sheet
- note S3. Modification of deconvolution protocol incorporating attenuation and attenuation-compensation
- note S4. Effect of incorrect attenuation estimation on deconvolution
- note S5. Determination of specimen attenuation
- note S6. Sample-based geometric effects on attenuation
- note S7. Attenuation-compensation of multiphoton excitation Airy and Bessel light sheets
- fig. S1. Pupil functions of attenuation-compensated Airy and Bessel beams.
- fig. S2. Look-up tables of achievable attenuation-compensation for Airy and Bessel beam parameters.
- fig. S3. Line profiles through simulated images of a 1D resolution target shown in Fig. 1 (main text).
- fig. S4. Simulated images: Effect on deconvolution from error in estimation of attenuation.
- fig. S5. Sample induced geometric effects on attenuation profile across the FOV.
- fig. S6. Attenuation-compensated LSM in a scattering sample.
- fig. S7. Local SBR and CNR measured for data shown in Fig. 4 (main text).
- fig. S8. Local SBR and CNR measured for data shown in Fig. 5 (main text).
- fig. S9. Light-sheet intensity profiles for the data shown in Fig. 5 (main text).
- fig. S10. Effect of attenuation and attenuation-compensation on two-photon excitation SPIM and DLSM Airy light sheet.

- fig. S11. Effect of attenuation and attenuation-compensation on two-photon excitation Bessel beam.
- fig. S12. Effect of attenuation and attenuation-compensation on two-photon excitation DSLM Bessel light sheet.
- fig. S13. Schematic of attenuation-compensated Airy light-sheet microscope.
- table S1. Experimental parameters for all data shown in main text.
- table S2. FOV of two-photon excitation SPIM and DLSM Airy light sheet with attenuation and attenuation-compensation.
- table S3. FOV of two-photon excitation Bessel beam and DLSM Bessel light sheet with attenuation and attenuation-compensation.

Supplementary Figures

Fig. #	Description	Page #
S1	Pupil functions of attenuation-compensated Airy and Bessel beams.	S14
S2	Look up tables of achievable attenuation-compensation for Airy and Bessel beam parameters.	S15
S3	Line profiles through simulated images of a 1D resolution target shown in Fig. 1 (Main Text).	S16
S4	Simulated images: Effect on deconvolution from error in estimation of attenuation.	S17
S5	Sample induced geometric effects on attenuation profile across the field-of-view.	S18
S6	Attenuation-compensated light-sheet microscopy in a scattering sample.	S19
S7	Local SBR and CNR measured for data shown in Fig. 4 (Main Text).	S20
S8	Local SBR and CNR measured for data shown in Fig. 5 (Main Text).	S21
S9	Light-sheet intensity profiles for the data shown in Fig. 5 (Main Text).	S23
S10	Effect of attenuation and attenuation-compensation on 2-photon excitation SPIM and DLSM Airy light-sheet.	S24
S11	Effect of attenuation and attenuation-compensation on 2-photon excitation Bessel beam.	S25
S12	Effect of attenuation and attenuation-compensation on 2-photon excitation DSLM Bessel light-sheet.	S26
S13	Schematic of attenuation-compensated Airy light-sheet microscope.	S27

S1 Attenuation-compensation of an Airy beam light-sheet

The following Note summarises the approach to attenuation compensation of Airy beams as described by Preciado, Dholakia, and Mazilu (28). This approach is expanded and discussed in the context of light-sheet microscopy. Throughout this discussion, we are consistent with the microscope coordinate system we have defined earlier, with the illumination propagation along the $x-$axis and optic axis of the detection optics along the $z-$axis.

Derivation of attenuation-compensation Airy field equation in linearly attenuating medium

The spatial Fourier spectrum of a 1+1D (1 lateral dimension + 1 longitudinal dimension) Airy beam propagating through a linearly absorbing medium can be approximated as

$$\tilde{E}(k_z, x) \approx \exp\left(\frac{ik_z^2 x}{2nk_0 - iC_{abs}} - ink_0 x - \frac{C_{abs}x}{2}\right)\exp\left(\frac{iz_0^3 k_z^3}{3}\right) \tag{S1}$$

where k_z is the spatial frequency of the transverse coordinate, z, x is the longitudinal coordinate, $k_0 = 2\pi/\lambda$ is the vacuum wave vector, z_0 is a scaling factor that dictates the extent of the Airy beam, and n and C_{abs} are the real refractive index and absorption coefficient of the absorbing medium respectively.

The Fourier transform of (S1) yields the propagation of the Airy field in the absorbing medium

$$E(z, x) = \text{Ai}\left(\frac{z}{z_0} - \frac{x^2}{4x_0^2}\right)\exp\left(-\frac{C_{abs}x}{2}\right)\exp\left(i\left[\frac{x^3}{12x_0^3} - \frac{x_0 x}{z_0^2} - \frac{zx}{2z_0x_0}\right]\right) \tag{S2}$$

where $x_0 = nk_0z_0^2$.

The middle term, $\exp(-C_{abs}x/2)$, characterises the decay of the field upon propagation. As this stems from a real exponential term in the Fourier spectrum of the beam, it can be accounted for by introducing another exponential, $\exp(-b_0k_z)$ which results in the field equation

$$\begin{aligned} E(z, x) = &\text{Ai}\left(\frac{z}{z_0} - \frac{x^2}{4x_0^2} + \frac{ib_0}{z_0}\right)\exp\left(-\frac{C_{abs}x}{2} + \frac{xb_0}{2z_0x_0}\right) \\ &\exp\left(i\left[\frac{x^3}{12x_0^3} - \frac{x_0 z}{z_0^2} - \frac{zx}{2z_0x_0}\right]\right) \end{aligned} \tag{S3}$$

The total intensity loss due to absorption, C'_{abs}, is now given by

$$C'_{abs} = C_{abs} - \frac{b_0}{z_0x_0} \tag{S4}$$

For $b_0 = z_0x_0C_{abs}$, the beam will completely counteract attenuation and the intensity of the main lobe will propagate without decay, as if in free-space. In free-space, $b_0 \neq 0$ will result in a beam where the intensity of the main lobe increases or decreases with propagation.

For a light-sheet with a cylindrical pupil function $P \equiv P(k_z)$ the above expressions are sufficient but this treatment can easily be extended to 2+1D Airy beams ($P \equiv P(k_z, k_y)$), where the overall compensation is the sum of the compensation along each individual axis

$$C'_{abs} = C_{abs} - \frac{b_{0z}}{z_0x_0} - \frac{b_{0y}}{y_0x_0} \tag{S5}$$

Attenuation-compensation is essentially the application of an amplitude mask in the pupil plane which weights each spectral component based on the path length of that component before it contributes to the main lobe of the beam. The above treatment considers only attenuation in a linearly absorbing medium ($C_{abs} = constant$), but it can be readily applied to complex systems where non-linear attenuation is encountered ($C_{abs} \equiv C_{abs}(x)$).

Ray optics approach to attenuation-compensation of Airy beam

Attenuation-compensation can be considered from a ray optics perspective as is shown in Fig. 1 (Main Text). The ray optics representation readily shows how the characteristic parabolic shape of the main caustic of the Airy beam arises from the cubic phase profile in the pupil plane. In

an attenuating medium, all the rays attenuate at equal rates but the rays which form the caustic furthest from the lens have travelled further than rays that form earlier parts of the caustic and have also therefore attenuated more at this point. Attenuation-compensation is achieved when the amplitude of each ray, at the point it forms the main caustic, is equal.

Normalised pupil function for an attenuation-compensated Airy light-sheet

The cylindrical pupil function of an attenuation-compensated Airy light-sheet is given by

$$P(u) = A_\sigma \exp(2\pi i \alpha u^3) \exp(-u^8) \exp(\sigma[u-1]) H(\sqrt{2} - |u|) \tag{S6}$$

where u in the normalised pupil coordinate corresponding to the $z-$axis, A_σ is a real scaling factor, α dictates the propagation-invariance of the Airy light-sheet (13), σ dictates the degree of linear attenuation-compensation, and $H(\cdot)$ denotes the Heaviside step function. The addition of the "soft-edge" 8th-order super-Gaussian apodization function (middle term in eq. (S6)) is used to eliminate sharp discontinuities which occur at the edge of the pupil function when attenuation-compensation is used and is consistent with the approach of Preciado, Dholakia, and Mazilu (28). To accommodate the "soft-edge" of this pupil function, the "hard-edge" apodization ($H(\cdot)$) is extended from $|u| = 1$ to $|u| = \sqrt{2}$. A_σ is chosen such that the maximum amplitude of the pupil function with any σ is unity and such that all light-sheets have the same peak intensity at the beginning of their field-of-view (FOV).

Experimentally, our light-sheet is formed either by cylindrical focussing of a 1+1D Airy beam with pupil function given by (S6) or by digital scanning of a spherically focussed 2+1D Airy beam (13) with pupil function given by:

$$\begin{aligned} P(u,v) =& A_\sigma \exp(2\pi i \alpha [u^3 + v^3]) \exp(-u^8) \exp(\sigma_u [u-1]) \\ & \exp(-v^8) \exp(\sigma_v [v-1]) H(\sqrt{2} - \sqrt{u^2+v^2}) \end{aligned} \tag{S7}$$

where the pupil function remains separable in Cartesian coordinates ($P(u,v) = P(u)P(v)$, except for minor contributions from $H(\cdot)$), and σ_u and σ_v denote modulation along the $u-$ and $v-$axes respectively.

Due to the separable nature of (S7) and the fact that the focussed beam is scanned along the $y - axis$ for formation of the light-sheet, the exact form of $P(v)$ is somewhat irrelevant to the cross-section of the light-sheet as long as the scan range is larger than the $y-$axis transverse extent of the beam. We therefore set $\sigma_v = 0$ in all further discussions and (S6) is an accurate description of the light-sheet pupil function. Modulation only compensates for attenuation of the light-sheet profile when applied along the $u-$axis. Figure S1 shows examples of the pupil functions given by (S6) and (S7).

Attenuation-compensated Airy light-sheet in terms of normalised pupil parameters

Considering the normalised pupil functions used to describe an Airy light-sheet, the transverse coordinate, k_z is related to the normalised transverse coordinate, u, by

$$u = k_z \frac{\lambda}{2\pi \mathrm{NA}} \tag{S8}$$

Similarly, z_0 and α, and b_0 and σ are related by the following equations

$$\alpha = \frac{1}{6\pi} \left(\frac{2\pi \mathrm{NA} z_0}{\lambda} \right)^3 \tag{S9}$$

$$\sigma = b_0 \frac{2\pi \mathrm{NA}}{\lambda} \tag{S10}$$

We denote the second term of (S4) χ, the degree of compensation. Re-expressed in terms of normalised coordinates this becomes

$$\chi = \frac{\sigma \mathrm{NA}^2}{3n\alpha\lambda} \tag{S11}$$

There is little that limits the degree of compensation that can be achieved for an Airy beam with a given $\alpha-$parameter other than the amount of power that can be delivered in the beam.

More practically for imaging, though, the extra energy delivered to deeper locations of the beam causes a non-negligible distortion of the beam profile. As the rays of the main caustic at greater depth influences the side-lobe structure earlier in propagation, the extra energy added to the beam causes the intensity of the side-lobes to increase. We define a practical limit for the maximum attenuation-compensation that can be applied as the degree of compensation at which the peak transverse intensity of the beam no longer follows the parabolic trajectory expected of the Airy beam within the longitudinal range set by the FOV of the Airy beam as given by

$$FOV_{Airy} = \frac{6\alpha\lambda}{n}\frac{1}{1-\sqrt{1-(\mathrm{NA}/n)^2}} \tag{S12}$$

Using these criteria, and simulations of beam profiles with various $\alpha-$ and $\sigma-$parameters, compensation must satisfy the following inequality

$$\alpha^{0.0635}\sigma \leq 0.6125 \tag{S13}$$

or expressed in real-space parameters

$$z_0^{3\times 0.0635} b_0 \leq 0.6125(6\pi)^{0.0635}\left(\frac{\lambda}{2\pi\mathrm{NA}}\right)^{(3\times 0.0635)+1} \tag{S14}$$

It is expected that different choices of "soft-edge" apodization in the pupil function will yield different results for the maximum permitted compensation, as well as small differences in the maximum achievable FOV.

Figure S2 shows the degree of compensation, χ, that can be achieved for $\alpha-$values commonly used for light-sheet microscopy (13). The lines of constant χ in Fig. S2(a,b) define a linear relation between σ and α. This is fundamentally linked to the relationship between the FOV and the $\alpha-$parameter of the Airy beam. If the FOV is doubled in a given linearly attenuating medium, the total attenuation across the FOV will also double, and so the $\sigma-$parameter will also have to be doubled to maintain full attenuation-compensation. The product of χ, for a given $\sigma-$parameter, and the FOV is a constant.

S2 Attenuation-compensation of a Bessel beam light-sheet

The following Note summarises the approach to attenuation-compensation of Bessel beams as described by Čižmár and Dholakia (26). Throughout this discussion, we are consistent with the microscope coordinate system we have defined earlier, with the illumination propagation along the $x-$axis and optic axis of the detection optics along the $z-$axis.

Derivation of method for producing Bessel beam with arbitrary axial envelope

The spatial Fourier transform of an azimuthally independent beam ($E(r, x = 0)$), such as a Bessel beam, can be expressed as a zero-order Hankel transform

$$\tilde{E}(k_r, x = 0) = \int_0^\infty E(r, x = 0) J_0(k_r r) r dr \tag{S15}$$

where $k_r = \sqrt{k_z^2 + k_y^2}$ is the wave vector of $r = \sqrt{z^2 + y^2}$, x is the longitudinal coordinate, and $J_0(k_r r)$ is the zero-order Bessel function.

It then follows by the inverse transform of (S15) that, for a Bessel beam, the on-axis propagation of the field ($E(r = 0, x)$) is linked with the radial mapping of the pupil ($\tilde{E}(k_r, x = 0)$) by:

$$E(r = 0, x) = \int_0^k \tilde{E}(k_r, x = 0) \exp(i k_x x) dk_x \tag{S16}$$

where $k_x = \sqrt{k_0^2 - k_r^2}$ and $k_0 = 2\pi/\lambda$ is the vacuum wave vector.

For a quasi-Bessel beam, $E_{Bessel}(r = 0, x) = A(x) \exp(i k_{x_0} x)$, where $A(x)$ is the desired axial envelope function, $k_{x_0} = \sqrt{k^2 - k_{r_0}^2}$, k_{r_0} is the radial wave vector corresponding to an ideal Bessel spectrum, the radial pupil function that can yield this envelope is found by the transform:

$$\tilde{E}(k_r, x = 0) = \frac{1}{2\pi k_x} \int_{-\infty}^{\infty} A(x) \exp(i k_{x_0} x) \exp(-i k_x x) dx \tag{S17}$$

A quasi-Bessel beam with a uniform intensity profile can be generated using a pupil function determined by solving (S17) with the axial envelope defined by

$$A_{uniform}(x) = \begin{cases} 1 & \text{if } |x| \leq x_{max} \\ 0 & \text{if } |x| > x_{max} \end{cases} \tag{S18}$$

where $2x_{max}$ is the maximum longitudinal extent of the Bessel beam.

To compensate for attenuation in a linearly absorbing medium, we have already established that the beam intensity must increase exponentially on propagation. Quasi-Bessel beams with exponentially increasing intensity on-axis can be generated from (S17) with the axial envelope defined by

$$A_{\sigma_B}(x) = \begin{cases} \exp(\sigma_B x / x_{max}) & \text{if } |x| \leq x_{max} \\ 0 & \text{if } |x| > x_{max} \end{cases} \tag{S19}$$

where σ_B is a constant that controls the degree of linear attenuation-compensation.

Consistent with the method described by Čižmár and Dholakia (26), and similarly to the method presented by Preciado, Dholakia, and Mazilu (28), a "soft-edge" apodization is applied to the determined pupil function in order to suppress high-frequency on-axis field oscillations which would otherwise distort the desired intensity profile. The apodization is a radial Gaussian centred on k_{r_0} with a width of $k_{r_0}/4$

$$\tilde{E}_{apodization}(k_r, x = 0) = \exp\left(\frac{-8(k_r - k_{r_0})^2}{k_{r_0}^2}\right) H\left(\sqrt{2} - k_r \frac{\lambda}{2\pi \text{NA}}\right) \tag{S20}$$

where $H(\cdot)$ is the Heaviside step function which limits the pupil to a finite extent and is consistent with our approach in Supp. Note S1.

Figure S1(e,f) shows numerically determined radial pupil function profiles for attenuation-compensated Bessel beams with various degrees of compensation.

Through numerical determination of the pupil function for attenuation-compensating Bessel beams and beam propagation simulations in linearly absorbing media, the relationship between compensation parameter, σ_B, and the degree of compensation, χ, was determined and is shown in Fig. S2. The lines of constant χ define an inverse relationship ($\sigma_B \propto \beta^{-1}$). This is consistent with the trend between α and σ observed in Fig. S2 as the FOV of a Bessel light-sheet scales inversely with β (13).

S3 Modification of deconvolution protocol incorporating attenuation and attenuation-compensation

The following Note describes modifications to the Airy light-sheet microscopy deconvolution strategy required to incorporate attenuation-compensated beams and attenuation. The original deconvolution strategy is described by Vettenburg *et al* (13).

Airy light-sheet microscopy deconvolution strategy

The recorded image stack can be considered as a convolution of the specimen fluorophore distribution with the light-sheet profile. The light-sheet profile dictates only the axial resolution but is spatially-variant along the propagation axis of the illumination. Therefore, a 1D spatially-variant Wiener filter is suitable to deconvolve the recorded image stack, given by

$$H_W(x,\nu_z) = \frac{H(x,\nu_z)^*}{|H(x,\nu_z)|^2 + SNR(\nu_z)^{-2}} \tag{S21}$$

where $H(x,\nu_z) = \mathcal{F}_z[LS(x,z)]$ is the 1D optical transfer function (OTF; Fourier transform) in z of the light-sheet profile, $LS(x,z)$, $SNR(\nu_z) = k\nu_{cut-off}/\nu_z$ is related to the signal-to-noise of the recorded images, modelled as a power law distribution, where k is a filter constant (typically $k = 5$) and $\nu_{cut-off} = 2\mathrm{NA}/\lambda$ where NA and λ are the numerical aperture and wavelength of the light-sheet respectively. To prevent edge artefacts in the deconvolution process, the light-sheet profile is zero-padded in z to double its axial dimension before calculation of the OTF.

The deconvolved image stack ($I_{dec}(x,y,z)$) is then determined by multiplication of the 1D Fourier transform along the $z-$axis of the image stack ($\tilde{I}_{rec}(x,y,\nu_z) = \mathcal{F}_z[I_{rec}(x,y,z)]$) with the Wiener filter ($H_W(x,\nu_z)$) and taking the 1D inverse Fourier transform along the $z-$axis to return this to real-space coordinates, given by

$$I_{dec}(x,y,z) = \mathcal{F}_{\nu_z}^{-1}[\tilde{I}_{rec}(x,y,\nu_z) \cdot H_W(x,\nu_z)] \tag{S22}$$

Again, before taking the Fourier transform of $I_{rec}(x,y,z)$, the first and last frames of the image stack are replicated in order to double its axial dimension and reduce the potential impact of deconvolution artefacts. After deconvolution, $I_{dec}(x,y,z)$ is cropped to the size of the original image stack to remove the replicated frames.

Determination of attenuation-compensated light-sheet profile

The only modifications required to deconvolve image stacks acquired with attenuation-compensated Airy light-sheets are the determination of the light-sheet profile, $LS(x,z)$, and the relative weighting of its OTF, $H(x,\nu_z)$.

The light-sheet profile, $LS(x,z)$, is determined by generating a 3D model of the attenuation-compensated Airy beam in the sample, and integrating this along the $y-$axis to yield the light-sheet profile. The beam profile is determined from the Fourier transform of the pupil function given by (S7) and propagated along the $x-$axis by multiplying the pupil function by a defocus term given by

$$P_{defocus}(u,v) = \exp(2\pi i d[u^2 + v^2]) \tag{S23}$$

where d is a defocus parameter related to the physical longitudinal displacement, Δx, by

$$d = \Delta x \frac{n}{\lambda}\left(1 - \sqrt{1 - \left(\frac{\mathrm{NA}}{n}\right)^2}\right) \tag{S24}$$

where n is the refractive index of the sample medium.

The light-sheet profile must be determined at $x-$ and $z-$coordinates corresponding to the $x-$ and $z-$pixel locations in the recorded image stack. As the beam profile is integrated along the $y-$axis to determine the light-sheet profile, accurate pixel-registration along the $y-$axis is not critical and can simply be Nyquist sampled.

The light-sheet profile is then modulated by a Gaussian envelope along the $z-$axis with width given by the depth-of-field (DOF) of the detection objective lens to suppress contributions from the light-sheet profile that generate fluorescence outwith the DOF and not contributing to signal.

To model attenuation, the light-sheet profile is further modulated by an exponential $(\exp(-C_{attn}^{D}x))$, where C_{attn}^{D} is the estimate of C_{attn} used in the deconvolution process.

The OTF of the attenuated light-sheet profile is then determined from the 1D Fourier transform along the $z-$axis as described above. This is typically normalised such that $|H(0,0)| = 1$. To account for the additional energy delivered by the light-sheet when using attenuation-compensation, the OTF was normalised such that $|H(0,0)| = 1$, and then multiplied by an additional scaling factor, $A_{OTF}(\sigma)$, given by

$$A_{OTF}(\sigma) = \frac{A_{\sigma}^{2}\exp(-2\sigma)}{A_{0}^{2}} \tag{S25}$$

where A_{σ} is a pupil amplitude scaling factor described in Supp. Note S1.

Deconvolution was then performed as described above using the weighted OTF.

S4 Effect of incorrect attenuation estimation on deconvolution

The following Note discusses the effects on the deconvolution of Airy LSM images caused by incorrect estimation of the sample attenuation.

In addition to determining the necessary degree of attenuation-compensation, precise knowledge of the attenuation profile is crucial for determining an accurate light-sheet PSF in an attenuating medium, which in turn is necessary for accurate deconvolution (see Supp. Note S3).

Here we consider error from the point of view of accurate deconvolution. Fig. 1 in the Main Text shows simulated images of the University Crest imaged with various degrees of attenuation-compensation in a linearly absorbing medium ($C_{attn} = 64.95\text{cm}^{-1}$). During the deconvolution process, the light-sheet profile as it would appear in free-space is modulated by an exponential function ($\exp(C^D_{attn}x)$) to model attenuation, where C^D_{attn} is the estimate of C_{attn} used for deconvolution. The images shown in Fig. 1 have been deconvolved with the correct attenuation coefficient ($C^D_{attn} = C_{attn} = 64.95\text{cm}^{-1}$). However, if the estimate for the attenuation coefficient, C^D_{attn}, deviates from the true attenuation, C_{attn}, then the deconvolved image will contain artefacts.

Figure S4 shows simulated images of the University Crest ($C_{attn} = 64.95\text{cm}^{-1}$) imaged with a standard ($\sigma = 0$; Fig. S4(a-g)) and attenuation-compensated ($\sigma = 0.54$; Fig. S4(h-n)) Airy light-sheet with an error in C^D_{attn} of $\pm 10\%$, $\pm 50\%$, and $\pm 100\%$ C_{attn}.

Figure S4 shows that the deconvolution artefacts resulting from incorrect estimation of specimen attenuation are essentially negligible if the magnitude of the error is below 10% C_{attn}. However, even in the absence of compensation, large underestimates of the sample attenuation shift the peak intensity towards the left of the image, whereas overestimates shift it towards the right. When C^D_{attn} matches the sample attenuation, the image intensity is fairly uniform across the FOV. This can be understood because the deconvolution utilises knowledge of the light-sheet PSF, including knowledge of its intensity as it propagates, to restore the sample fluorophore distribution.

Errors in C^D_{attn} also introduce errors in the estimate of the light-sheet OTF. As discussed in the Main Text, attenuation of the Airy light-sheet reduces the MTF, and therefore the maximal achievable axial resolution. If the attenuation is underestimated, the MTF will be overestimated, and noise will be amplified as the light-sheet propagates (Fig. S4(a-c,h-j)). If the attenuation is overestimated, the MTF will be underestimated, and high spatial frequency details will be rejected from the image as noise. Underestimation of the MTF effectively leads to excessive axial low-pass filtering of the image, as observed in Fig. S4(e-g,l-n).

S5 Determination of specimen attenuation

The following Note discusses strategies for determining the attenuation of a specimen in order to achieve effective and correct attenuation-compensation.

In the attenuating phantom seeded sparsely with fluorescent beads (Fig. 3), the background is weakly fluorescent. A mean projection over a data volume will be dominated by the background signal, which decays with attenuation and therefore can be accurately fit to give the absorption coefficient.

In a non-fluorescent attenuating phantom or a biological specimen (Fig. 4 - 6), the signal as a function of depth in the data cube will depend on the density and distribution of fluorophores as well as the attenuation, so a mean projection cannot be used to estimate the absorption coefficient. We found that it was necessary to perform a maximum value projection and estimate the attenuation on a per-image basis. Depending on the uniformity of the sample, it may be necessary to perform this procedure per-image or simply per-sample. We assumed exponential intensity decay across the image, and divided the image by a decaying exponential as a function of $x-$axis coordinate. The attenuation was determined to be the decay parameter which yielded approximately uniform intensity structures throughout the image after division.

S6 Sample-based geometric effects on attenuation

The following Note discusses the effects of specimen geometry on the attenuation profile of the light-sheet.

In a microscope with collinear illumination and detection, the intensity of fluorescence signal at any point in the specimen is given by the Beer-Lambert law

$$I_{Fluor}(r) = \exp[-C_{attn}r] \tag{S26}$$

where C_{attn} is the attenuation coefficient, which we assume to be constant for simplicity, and r is the specimen thickness (in transillumination) or the imaging depth (in epi-illumination).

In the case of light-sheet microscopy, the orthogonal illumination and detection pose additional geometric challenges for attenuating specimens. The observed attenuation profile will be the product of the light-sheet attenuation profile and the spatially-variant attenuation of the generated fluorescence.

The simplest case is a sample with a square cross section, with illumination and detection pathways parallel to two sides of the square (Fig. S5(a)). The attenuation profile of the light-sheet is exponential on propagation, and the fluorescence attenuation profile is constant across the FOV. Therefore the fluorescence intensity is given by

$$I_{Fluor}(x) \propto \exp[-C_{attn}x] \tag{S27}$$

In the case of tissue slices oriented at 45° to both illumination and detection pathways (Fig. S5(b)), the attenuation of the fluorescence signal at any point across the FOV is equal to the attenuation of the light-sheet at that point by symmetry. Therefore the total fluorescence intensity is given by

$$I_{Fluor}(x) = \exp[-2C_{attn}x] \tag{S28}$$

Embryonic or spheroidal specimens will have a circular cross section (Fig. S5(c)). The flat plane of the light-sheet and the circular edge of the specimen result in the spatially-variant fluorescence intensity given by

$$I_{Fluor}(x) = \exp[-C_{attn}(x + R\sin(\pi x/2R))] \tag{S29}$$

where R is the radius of the specimen cross section.

Figure S5(d) summarizes the profiles given by (S27) - (S29). The additional optical path length when imaging in tissue sections causes a more rapid attenuation of the fluorescence across the FOV, as if the attenuation has effectively doubled). The morphology of spheroidal tissue results in a more complex attenuation profile. As the light-sheet propagates from the initial edge of the specimen (x_0) to its centre the attenuation is rapid (approximating an exponential decay with coefficient greater than twice the standard specimen attenuation coefficient). From the centre to the far edge, the rate of attenuation decreases, and the intensity even increases slightly at the far edge.

In this study, we have only simulated attenuation according to the linear, constant-attenuation model in equation (S27). We have used the linear constant-attenuation profiles (S27) and (S28) to model attenuation in the specimens discussed in the Main Text. The fluorescent beads in attenuating phantom (Section 2.2) were prepared in a glass square-walled capillary tube and oriented as in S5(a). Attenuation in the mouse brain tissue slice discussed in Section 2.3 was described by eq. (S28). The *S. lamarcki* opercula imaged in Section 2.3 can be considered 'quasi-circular' in cross-section but since the effective radius is larger than the FOV of the microscope, it could also be modelled using eq. (S27) rather than eq.(S29). However, the principle of attenuation is more general than linear attenuation and can be used to counteract any decay profile. Indeed, an important future step will be to determine accurate real-time methods to model and correct for arbitrary decay profiles in dynamic specimens.

S7 Attenuation-compensation of multi-photon excitation Airy and Bessel light-sheets

The following Note discusses the attenuation and attenuation-compensation of two-photon excitation (2PE) schemes in Airy and Bessel beam-based light-sheet imaging.

Using the framework for attenuation-compensation described in Supp. Notes S1 and S2, we extend our analysis to consider the effects of attenuation and attenuation-compensation on 2PE light-sheet microscopy. 2PE is a non-linear optical process, and its efficiency scales with the square of the illumination intensity. As highlighted in the Main Text, there are differences in the performance of Bessel beam light-sheet imaging modes dependent on whether the beam is swept rapidly or discretely stepped. In the non-linear regime, there are also subtle differences in the generation of an Airy light-sheet, dependent on whether a 1+1D Airy beam is used in conjunction with a cylindrical lens, or if a 2+1D Airy beam is used in conjunction with rapid, single-axis scanning. Here, we consider all 4 scenarios for completeness.

The 2PE cross-section of an Airy light-sheet formed by cylindrical focusing of a 1+1D Airy beam depends on the intensity distribution of the illuminating light-sheet. By contrast, when the light-sheet is formed by digital scanning of a 2+1D Airy beam, the 2PE cross-section is defined by the intensity distribution of the beam. Scanning of this 2PE beam cross-section gives the 2PE cross-section of the light-sheet as a whole. Likewise, the 2PE cross-section of a stepped Bessel beam is simply the square of the intensity of the illuminating Bessel beam, while the 2PE cross-section of a digitally scanned Bessel beam light-sheet is the integral of the 2PE beam cross-section along the scan axis.

For each scenario, the relevant beam or light-sheet profile was simulated for the centre wavelength of the illumination (800nm); the effect of attenuation (absorption) was applied to the illumination; and then the 2PE cross-section was determined from the resulting intensity profile. Beam parameters were adjusted to give equivalent FOV and attenuation-compensation properties to the single-photon excitation simulations shown in earlier Sections. In all four cases, the effect of under-compensated attenuation has a much more dramatic effect on the effective FOV of the 2PE signal due to the non-linear dependence of the 2PE cross-section on illumination intensity, as summarized in Tables S2 and S3.

2PE Airy light-sheet microscopy with a 1+1D Airy SPIM

Figure S10(a-d) show the intensity profiles of 1+1D Airy light-sheets with a centre wavelength of 800nm, $\alpha = 5$, and various degrees of attenuation-compensation. For this light-sheet, $\sigma = 0.57$ allowed compensation of $C_{abs} = 65\text{cm}^{-1}$ attenuation. Figure S10(f-i) show the associated 2PE cross-sections for these light-sheets, and Fig. S10(e,j) show the transverse peak intensity of the light-sheets on propagation.

2PE Airy light-sheet microscopy with a 2+1D Airy DSLM

Figure S10(k-n) show the 2PE cross-sections of light-sheets formed by digital scanning. The parameters are the same as for as their cylindrically focused counterparts shown in Fig. S10(f-i). The illumination intensity profiles are the same for both light-sheet generation methods (Fig. S10(a-d)). Like the 1+1D 2PE Airy light-sheet, the FOV of the 2+1D light-sheet is severely affected by under-compensation (Fig. S10(o)).

2PE Bessel beam light-sheet microscopy with a stepped Bessel beam

Figure S11(a-d) shows the intensity profile of the illuminating, stepped Bessel beam with centre wavelength of 800nm, $\beta = 0.75$, and various degrees of attenuation-compensation. For this beam, $\sigma_B = 0.22$ allowed compensation of $C_{abs} = 65\text{cm}^{-1}$ attenuation. Figure S11(f-i) shows the associated 2PE cross-sections of these beams, and Fig. S11(e,j) show the on-axis profiles of these beams on propagation. Again, the effect of under-compensation of attenuation is more pronounced on the 2PE cross-section than on the illumination profile.

2PE Bessel beam light-sheet microscopy with a digitally scanned Bessel beam

The 2PE cross-section of a digitally scanned Bessel beam light-sheet is the integral of the 2PE beam cross-section along the scan axis (Fig. S12(a-d)). Figure S12(f-i) shows the 2PE light-sheet cross-sections of the Bessel beams shown in (a-d), and Fig. S12(e,j) show the on-axis profiles of these light-sheets on propagation.

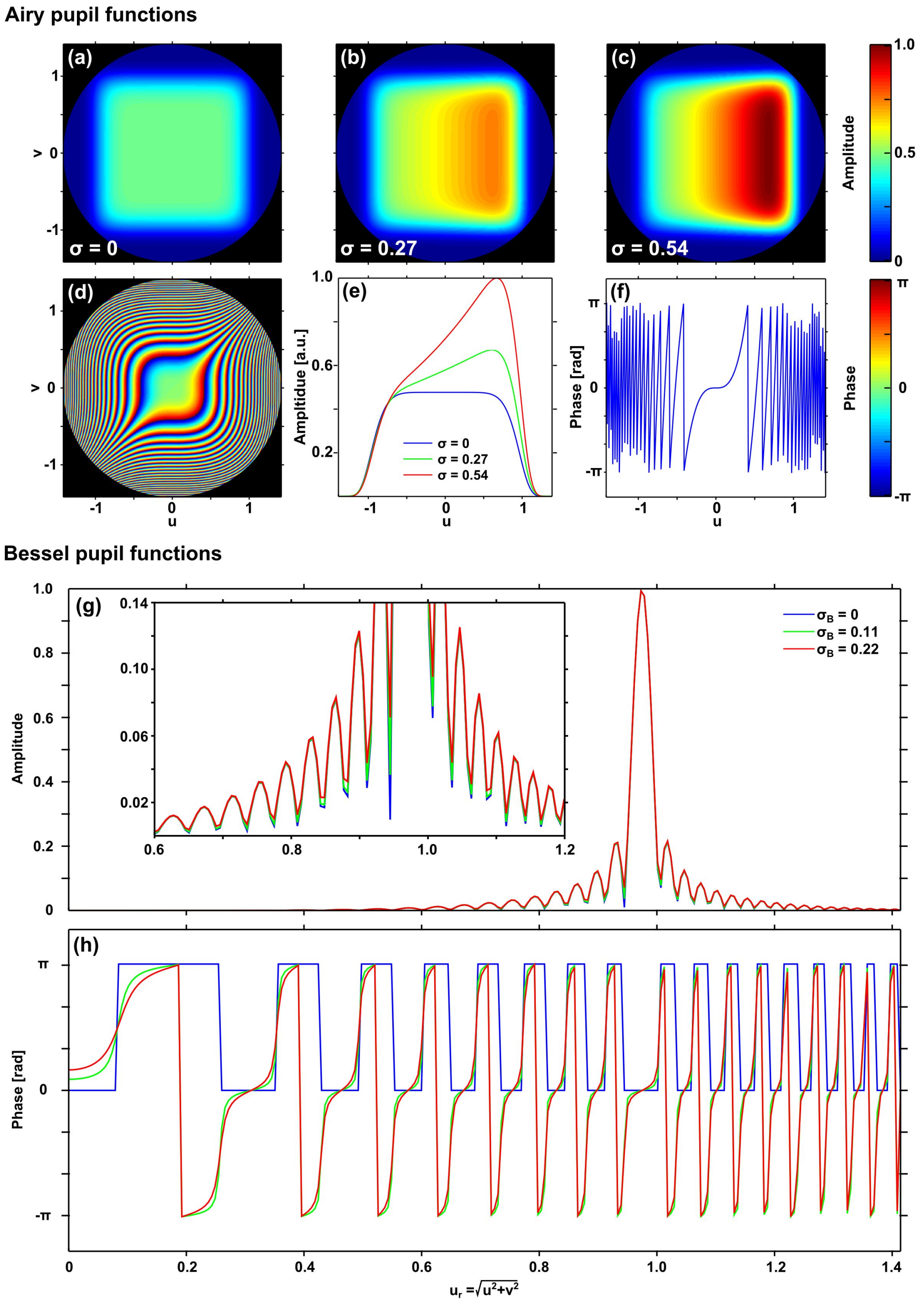

fig. S1. Spherical attenuation-compensated Airy beam ($\alpha = 7$) amplitude (a-c) and phase (d) pupil functions and their cylindrical (e,f) counterparts respectively. For all σ, the phase profile (d,f) is unaffected. Amplitude (g) and phase (h) of radial pupil functions for attenuation-compensated Bessel beams ($\beta = 0.05$). Inset shows finer detail of amplitude structure. Bessel pupil amplitudes in (g) have been normalised to their maximum value for display. See Supp. Notes S1 and S2 for more details on the pupil function parameters.

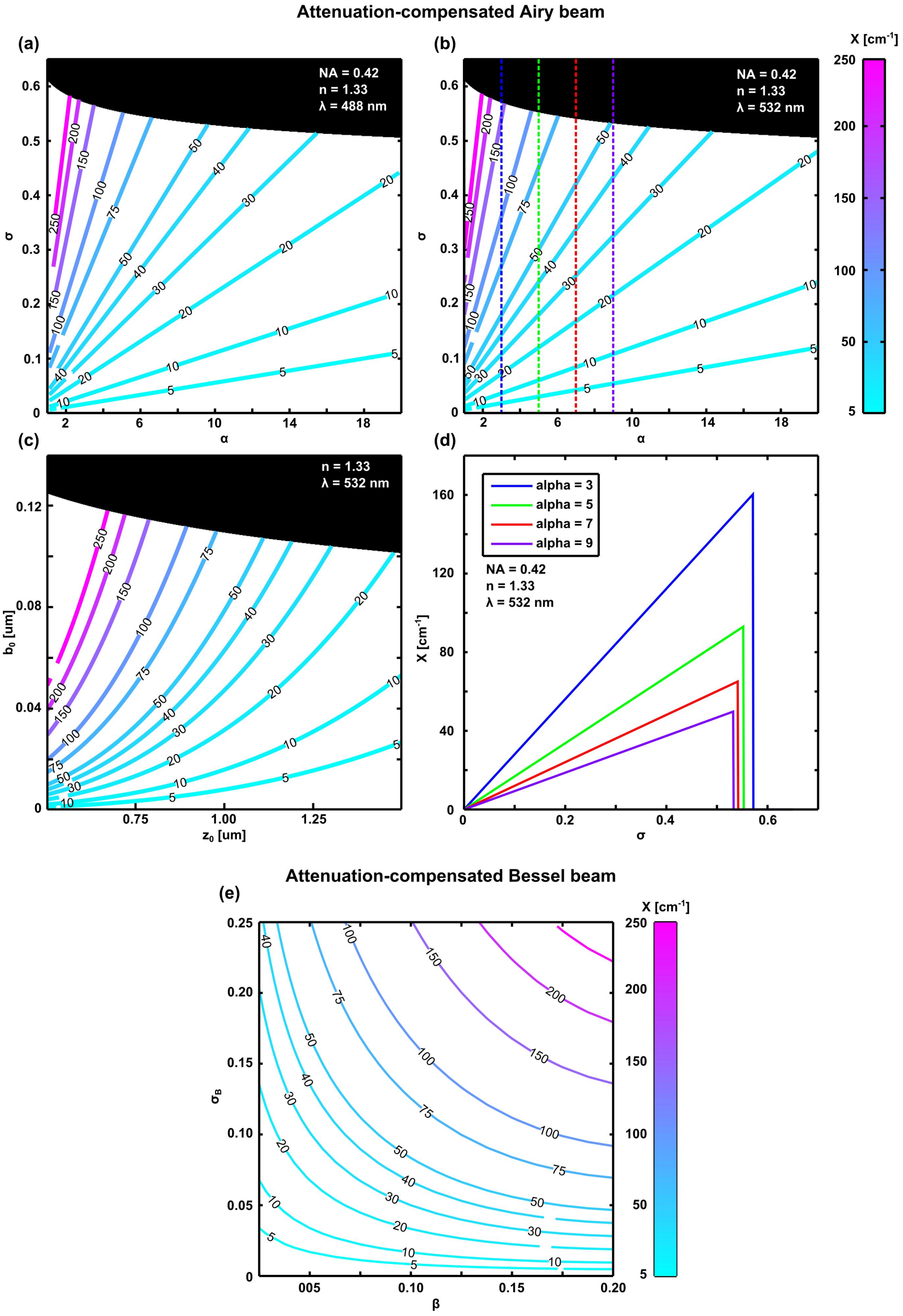

fig. S2. Look up tables for attenuation-compensated Airy light-sheet. Contour plot showing lines of constant χ for combinations of $\alpha-$ and $\sigma-$values for (a) $\lambda = 488$ nm and (b) $\lambda = 532$ nm. (c) Same plot as (b) but expressed in terms of real-space parameters, z_0 and b_0. (d) lines profiles taken through (b) for selected $\alpha-$values. Black regions in (a-c) indicate forbidden regions not satisfying the limits defined in (S13) and (S14). (e) Look up table for compensated Bessel beam. Contour plot showing lines of constant χ for combinations of $\beta-$ and σ_B-values for $\lambda = 532$ nm, NA $= 0.42$, $n = 1.33$.

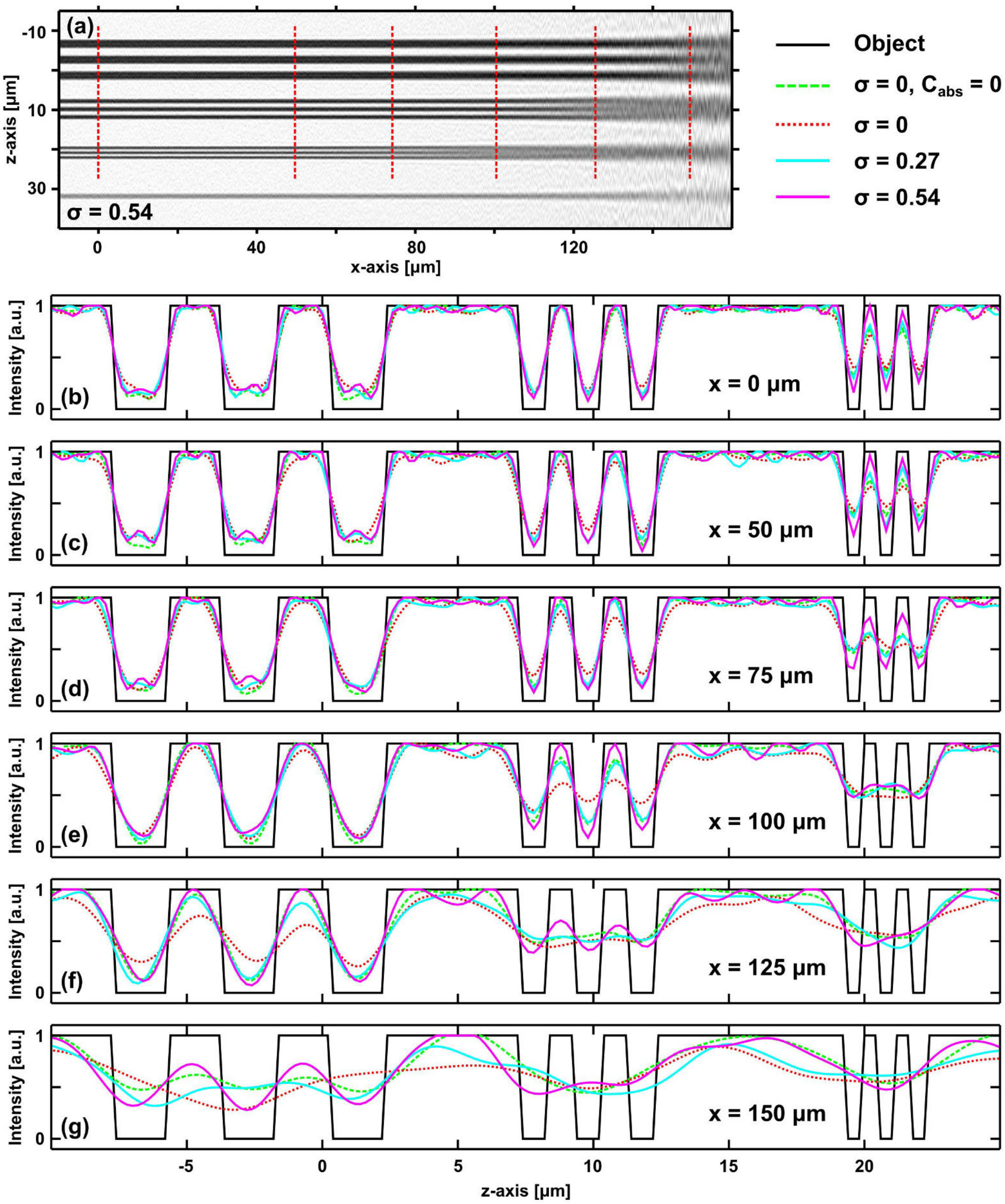

fig. S3. Intensity profiles through simulated Airy LSM images of the 1D resolution target shown in Fig. 1(s - u). Intensity profiles are taken at the locations shown in (a). (b) $x = 0\,\mu\text{m}$, (c) $x = 50\,\mu\text{m}$, (d) $x = 75\,\mu\text{m}$, (e) $x = 100\,\mu\text{m}$, (f) $x = 125\,\mu\text{m}$, (g) $x = 150\,\mu\text{m}$. Line width/spacing: $2\,\mu\text{m}$ (top), $1\,\mu\text{m}$, $0.6\,\mu\text{m}$, $0.2\,\mu\text{m}$ (bottom).

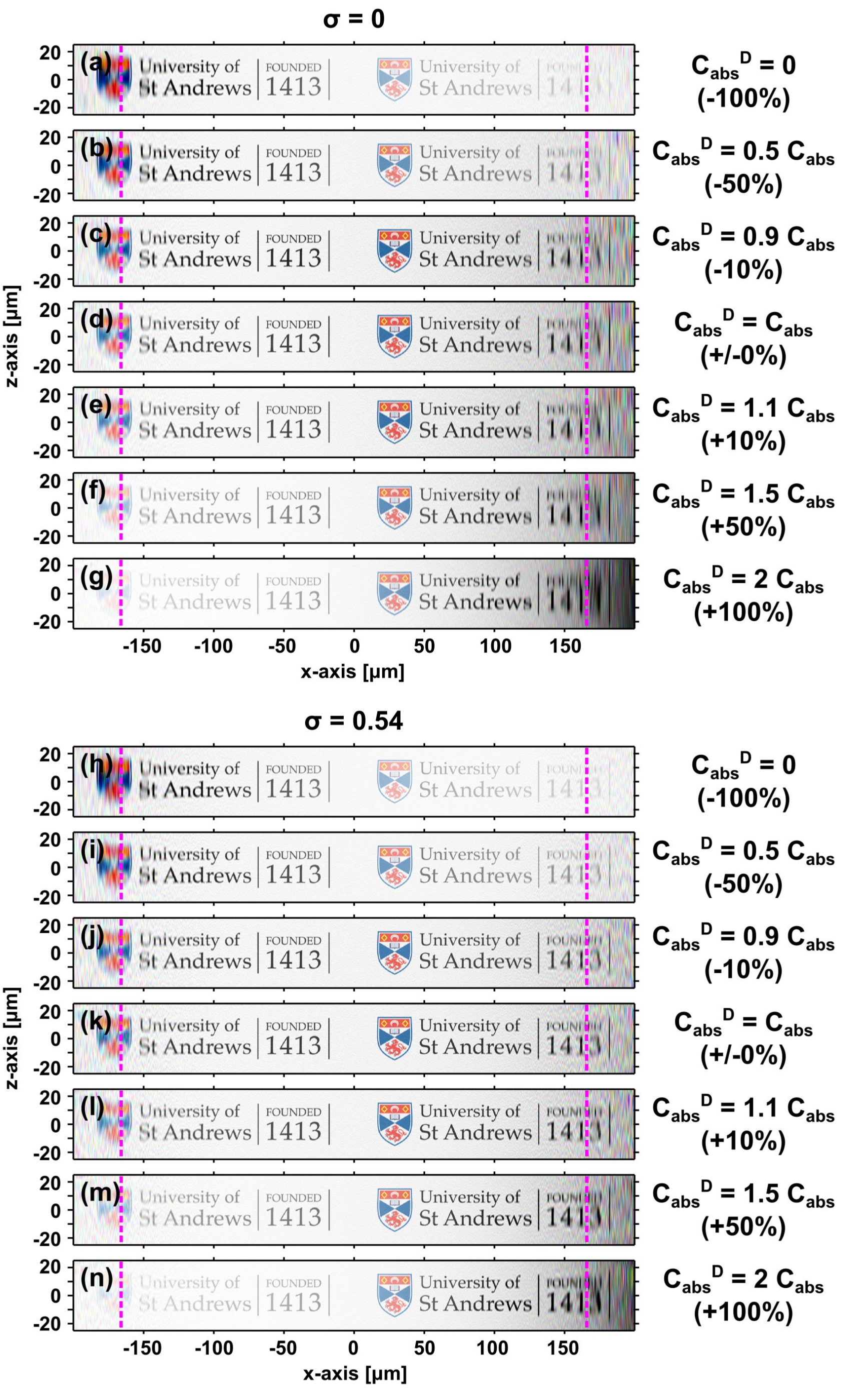

fig. S4. Simulated images of University Crest imaged by standard ($\sigma = 0$; (a-g)) and attenuation-compensated ($\sigma = 0.54$; (h-n)) Airy LSM in absorbing medium ($C_{abs} = 64.95\text{cm}^{-1}$) with ±10%, ±50%, and ±100% error in the estimation of the sample attenuation.

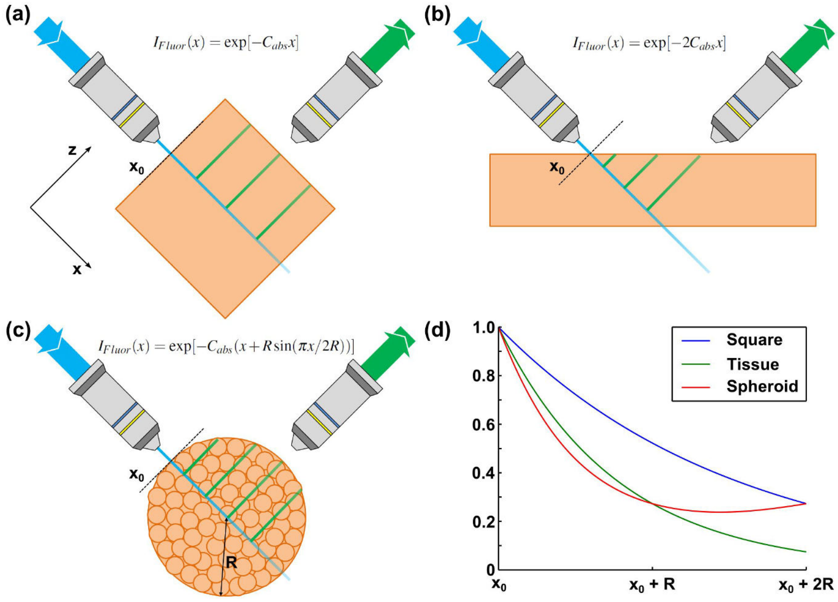

fig. S5. Illustrations of different sample geometries in LSM (a-c) and the intensity decay expected across the image for each geometry (d).

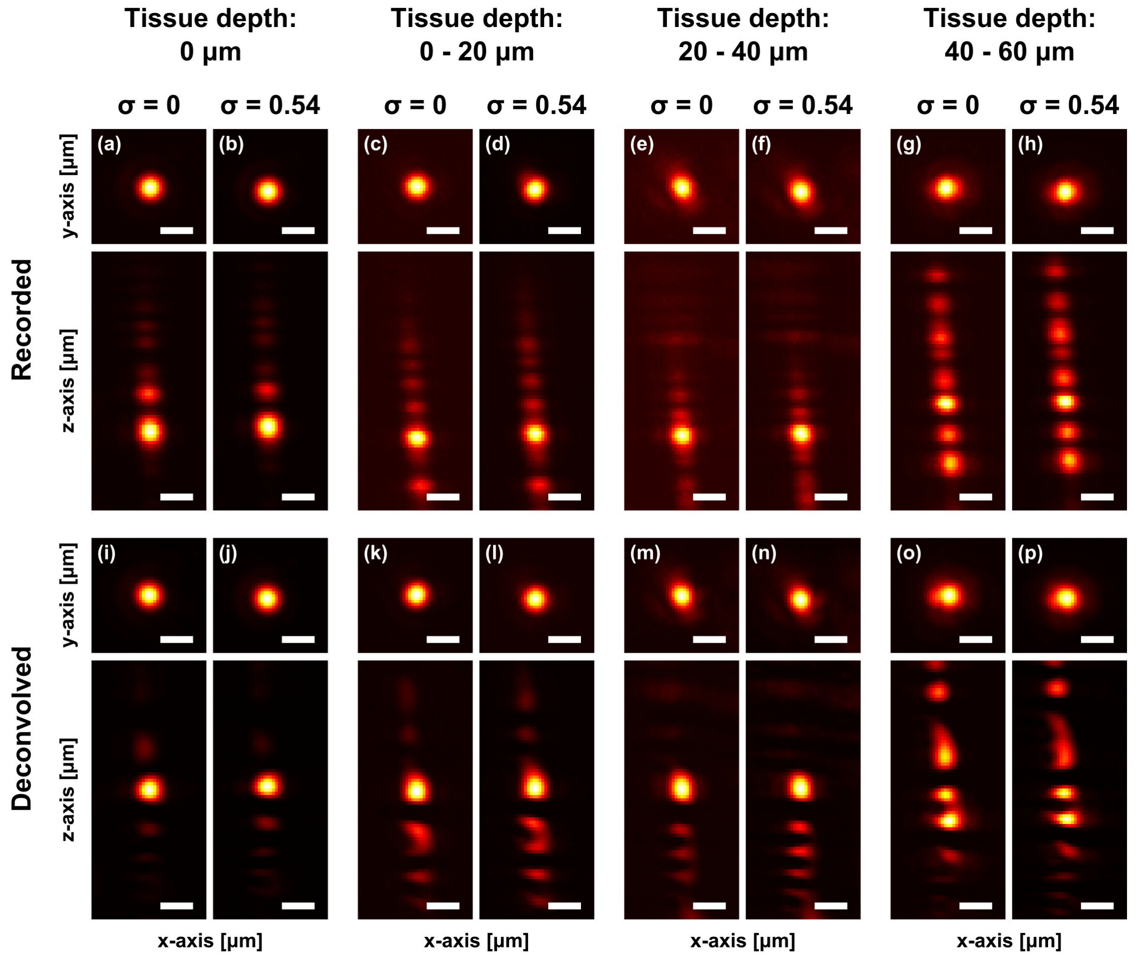

fig. S6. Attenuation-compensated Airy light-sheet microscopy in a scattering sample. Isolated sub-diffraction limited fluorescent microspheres imaged through a section of mouse brain tissue. Maximum intensity projections ($x-y$: top, $x-z$: bottom) of recorded data (a-h) and deconvolved images (i-p) for light-sheets with no attenuation-compensation ($\sigma = 0$; a, c, e, g, i, k, m, o) and with maximum attenuation ($\sigma = 0.54$; b, d, f, h, j, l, n, p). Beads were illuminated through various thicknesses of mouse brain tissue - (a, b, i, j) No tissue, (c, d, k, l) $0-20\,\mu$m thickness, (e, f, m, n) $20-40\,\mu$m thickness, (g, h, o, p) $40-60\,\mu$m thickness. While the quality of PSF decreases with increasing tissue thickness, the quality with and without attenuation-compensation for a given thickness is effectively unchanged. Scale bar: $2\,\mu$m.

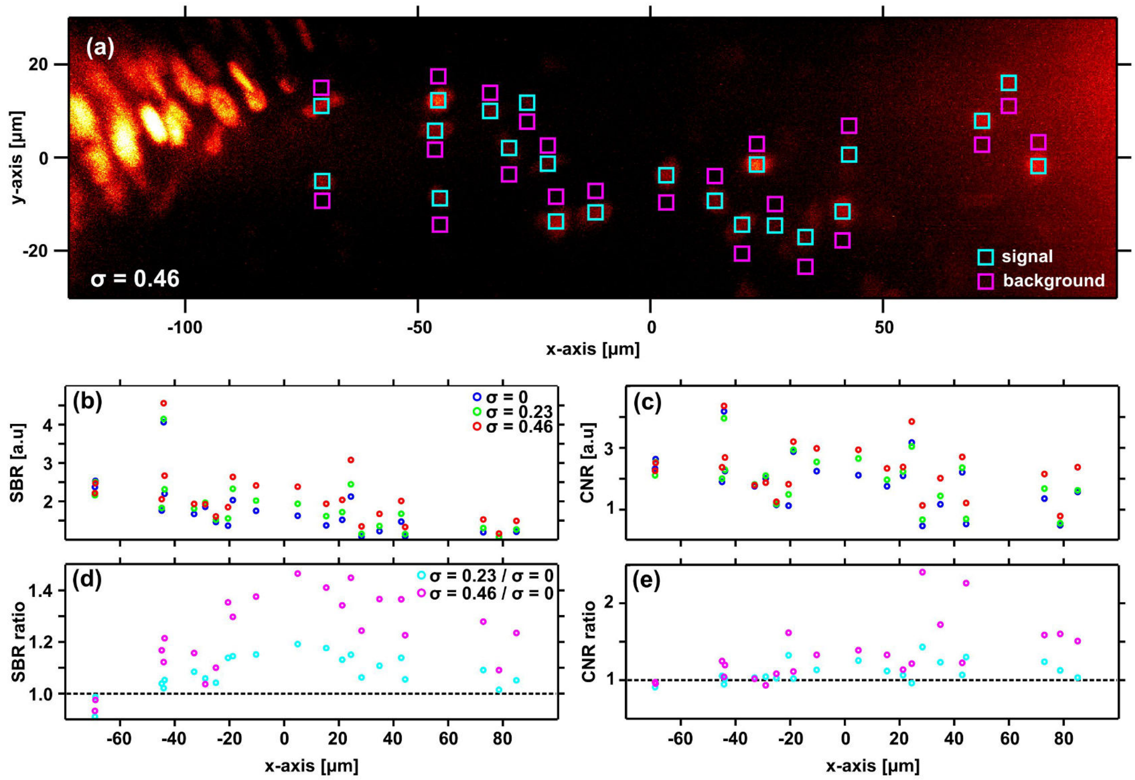

fig. S7. (a) Maximum intensity projection of deconvolved Airy LSM image of nuclei (propidium iodide) in the operculum of *S. lamarcki* (attenuation estimated at 85cm^{-1}) with $\sigma = 0.46$ as shown in Fig. 3. $3\times3\,\mu\text{m}^2$ signal regions are highlighted by cyan squares on identified nuclei. Background regions were selected in close proximity, and on the same $x-$axis coordinate, to identified nuclei as shown by magenta squares. (b,c) Local signal-to-background ratio (SBR) and contrast-to-noise ratio (CNR) plotted as a function of propagation coordinate, x, for $\sigma = 0$ (blue), $\sigma = 0.23$ (green), and $\sigma = 0.46$ (red). (d,e) Ratios of SBR and CNR with $\sigma = 0.23$ (cyan) and $\sigma = 0.46$ (magenta) to $\sigma = 0$. Even with partial attenuation-compensation, an improvement in the SBR and CNR is observed. With $\sigma = 0.23$, the effective attenuation after compensation is $C'_{attn} = 57.5\text{cm}^{-1}$ and an improvement of between $5-20\%$ in SBR and $5-50\%$ in CNR is observed. With $\sigma = 0.46$, the effective attenuation after compensation is $C'_{attn} = 30\text{cm}^{-1}$ and an improvement of between $20-45\%$ in SBR and $20-140\%$ in CNR is observed.

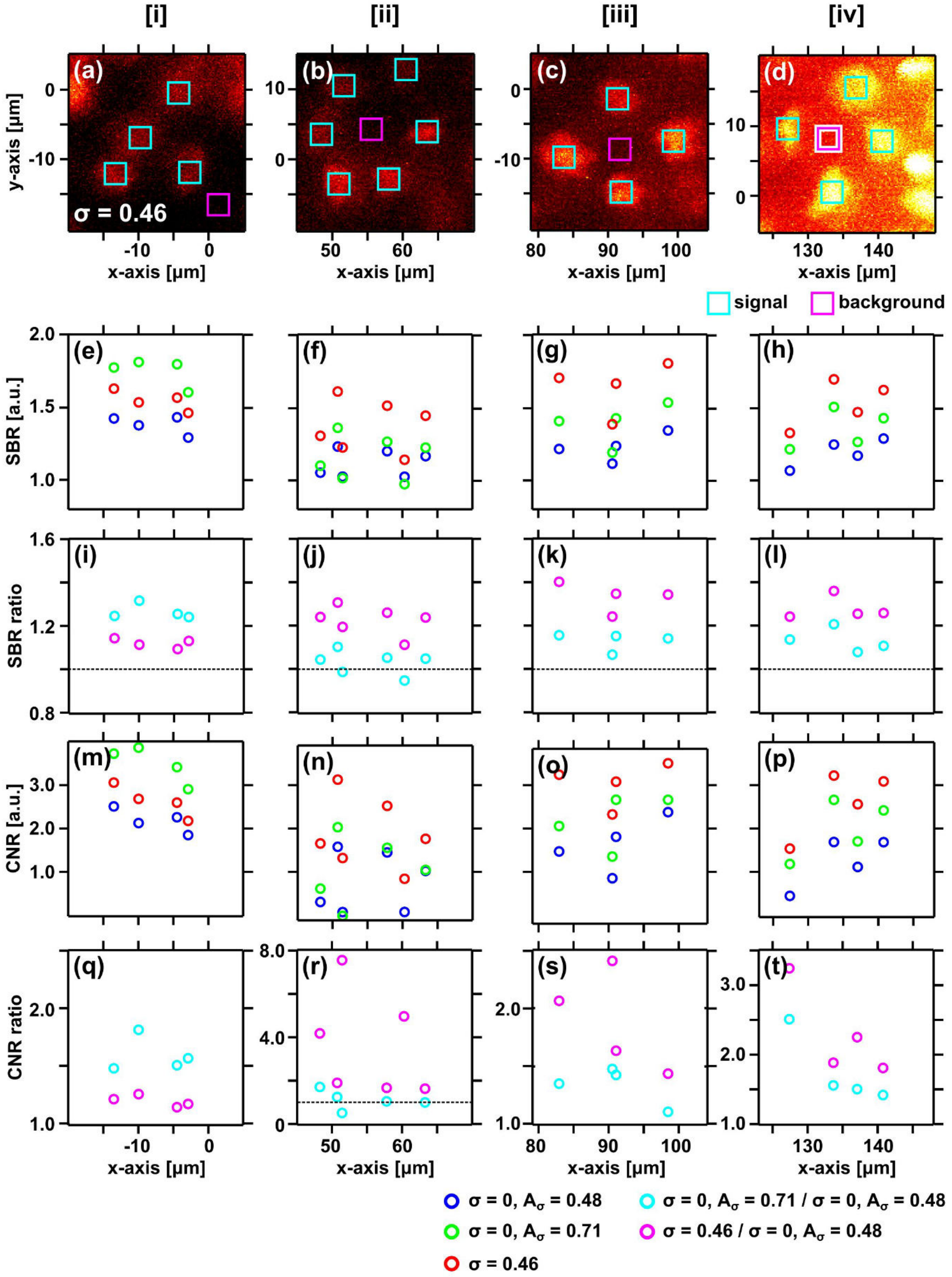

fig. S8. (a-d) Maximum intensity projection of deconvolved Airy LSM image of nuclei (propidium iodide) in the operculum of *S. lamarcki* (attenuation estimated at 75cm^{-1}) with $\sigma = 0.46$ as shown in Fig. 4. Regions [i]-[iv] indicate the same regions highlighted in Fig. 4. $3 \times 3\,\mu\text{m}^2$ signal regions are highlighted by cyan squares on identified nuclei. Background regions were selected in close proximity to identified nuclei as shown by magenta squares. The same background region was used for all signal regions within the ROI. (e-h) Local signal-to-background ratio (SBR) plotted as a function of propagation coordinate, x, for $\sigma = 0$, $A_\sigma = 0.48$ (blue), $\sigma = 0$, $A_\sigma = 0.71$ (green), and $\sigma = 0.46$ (red). (i-l) Ratios of SBR with $\sigma = 0$, $A_\sigma = 0.71$ (cyan) and $\sigma = 0.46$ (magenta) to (continued on next page)

Figure S8: (continued from previous page) $\sigma = 0$, $A_\sigma = 0.48$. In regions [ii]-[iv], the use of compensation (red/magenta) increases the SBR by $20-40\%$ whereas increasing the power without attenuation-compensation (green/cyan) increased the SBR by only $0-20\%$. In region [i], increasing the power without attenuation-compensation improves the SBR more than the use of attenuation-compensation but this is expected given the significant increase in intensity in the non-compensated light-sheet at this point (see Fig. S9). (m-p) local contrast-to-noise ratio (CNR) plotted as a function of propagation coordinate for the same illumination schemes and (q-t) the ratios of CNR with $\sigma = 0$, $A_\sigma = 0.71$ (cyan) and $\sigma = 0.46$ (magenta) to $\sigma = 0$. At $x \approx 60\,\mu\text{m}$ a datapoint for the case of $\sigma = 0$, $A_\sigma = 0.71$ (green.cyan) is missing from (n,r). The CNR for this feature was negative, owing to low signal, and therefore the CNR ratio relative to the case of $\sigma = 0$, $A_\sigma = 0.48$ is ill defined. The same trend is observed for CNR as for SBR. In regions [ii]-[iv] the use of compensation increased the CNR by $50 - 650\%$.

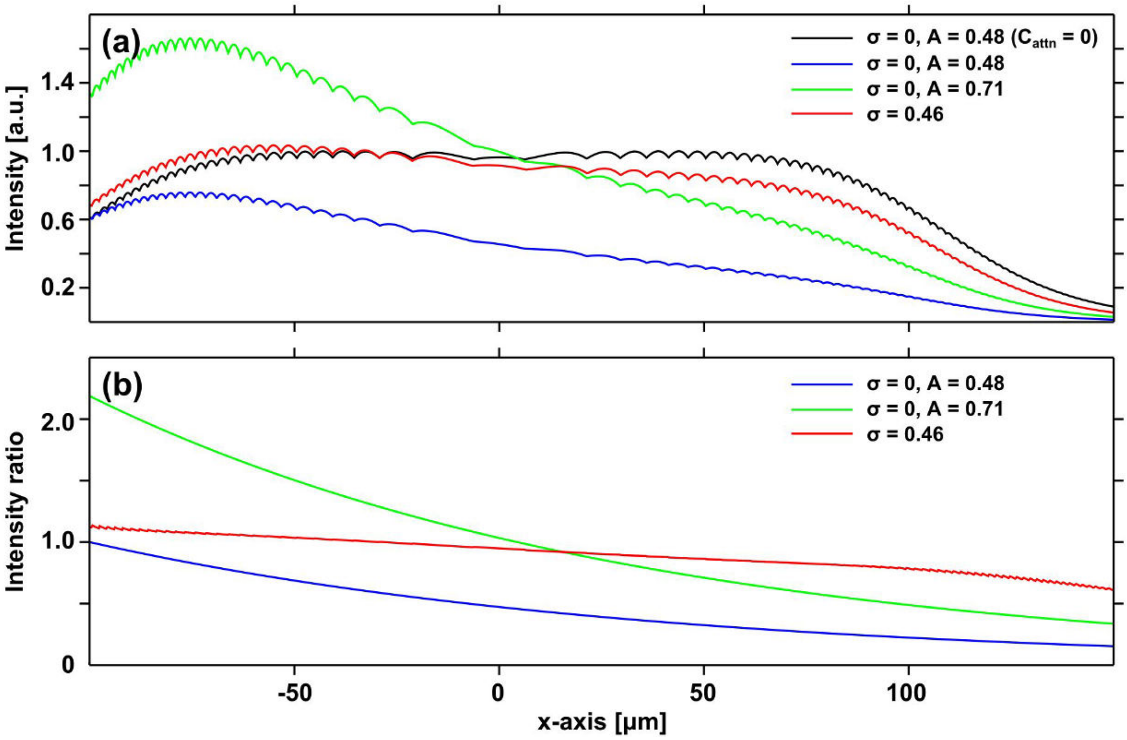

fig. S9. (a) The integrated intensity profiles of simulated Airy light-sheets as a function of propagation distance as used for deconvolution in Fig. 4. Black: uncompensated, unattenuated (ideal) light-sheet ($\sigma = 0$, $C_{attn} = 0$). Blue: uncompensated light-sheet with $C_{attn} = 75\text{cm}^{-1}$ applied. Red: compensated light-sheet with $\sigma = 0.46$ and $C_{attn} = 75\text{cm}^{-1}$. Green: an uncompensated light-sheet with $C_{attn} = 0$ and $\sigma = 0$, and the amplitude factor increased to $A_\sigma = 0.71$ such that its total power is equal to that of the compensated light-sheet (red). (b) Ratios of the attenuated and/or compensated light-sheet profiles to the ideal light-sheet (black curve in (a)). Blue: the attenuated, uncompensated light-sheet decays exponentially with propagation distance. Green: the peak power delivered by the attenuated, high-power light-sheet is more than twice the power delivered by the ideal light-sheet. However, it also decays exponentially into the medium. Red: due to strategic redistribution of power, the peak power delivered by the compensated light-sheet is never more than 10% greater than the power of the original, even though its power at the back aperture is equal to that of the high-powered beam (green). Its intensity profile is much flatter, and it stays within 10% of the intensity of the ideal beam over a distance of about 150 μm.

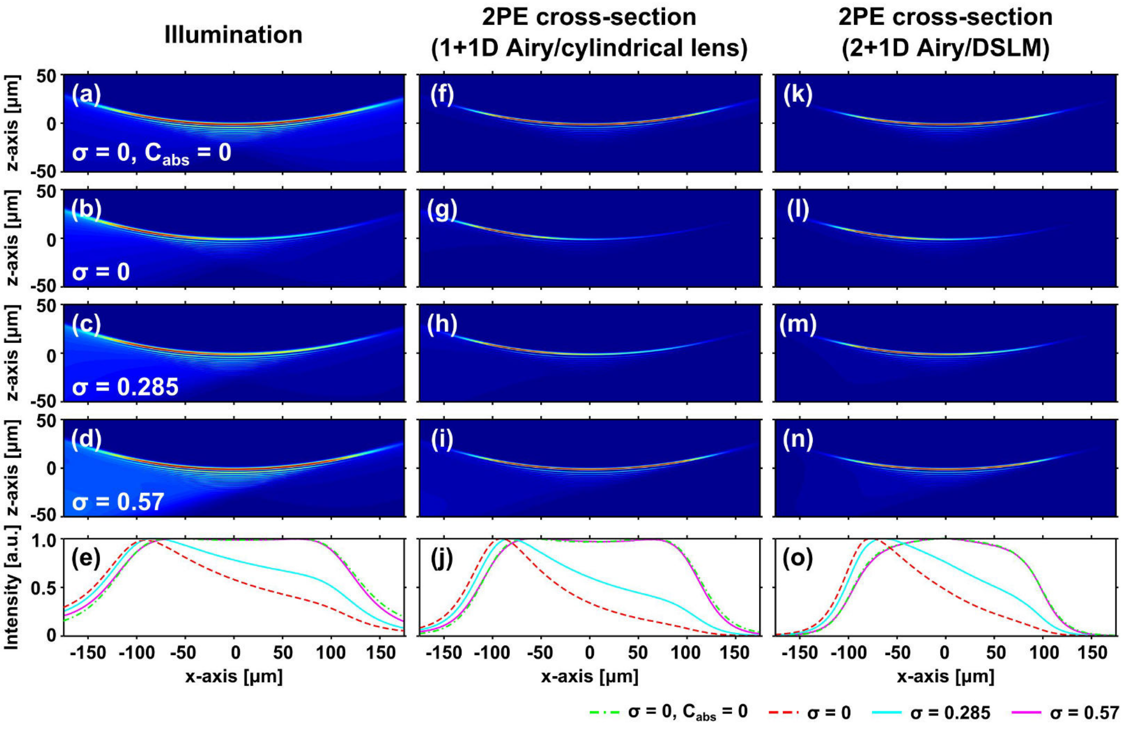

fig. S10. Illumination profiles (a-d) of attenuation-compensated 2PE Airy light-sheets and their corresponding 2PE cross-sections for light-sheets formed by a 1+1D Airy beam and a cylindrical lens (f-i) and by a 2+1D Airy beam and digital scanning (k-n) for no attenuation and no compensation (top row), attenuation ($C_{abs} = 65\text{cm}^{-1}$) and no compensation (2nd row), partial compensation (3rd row), and full compensation (4th row). Peak transverse intensity profiles for the illumination, and 2PE cross sections are shown in (e,j,o) respectively. The illumination cross-section (a-e) does not depend on the method of light-sheet generation.

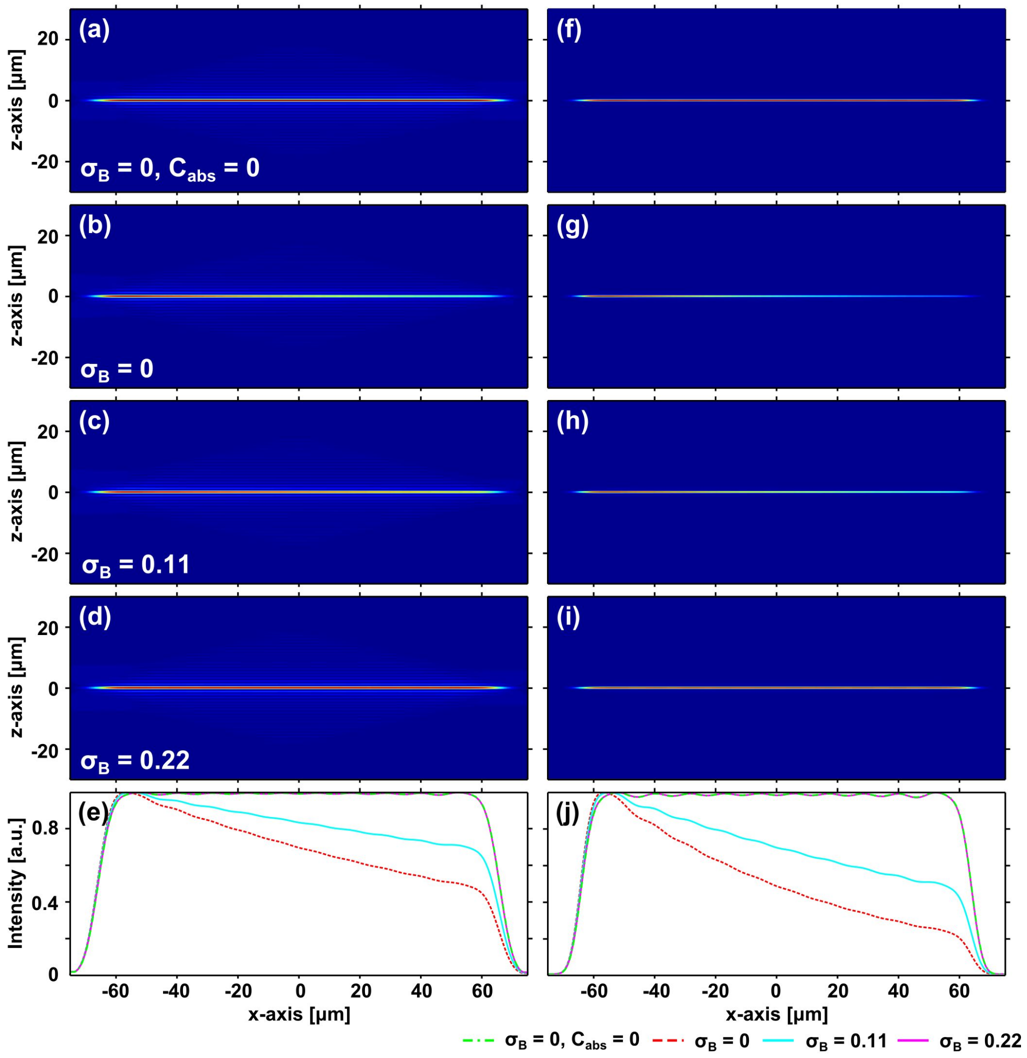

fig. S11. Illumination profiles (a-d) of attenuation-compensated 2PE Bessel beams and their corresponding 2PE cross-sections (f-i) for no attenuation and no compensation (top row), attenuation ($C_{abs} = 65\text{cm}^{-1}$) and no compensation (2nd row), partial compensation (3rd row), and full compensation (4th row). On-axis intensity profiles for the illumination, and 2PE cross sections are shown in (e,j) respectively.

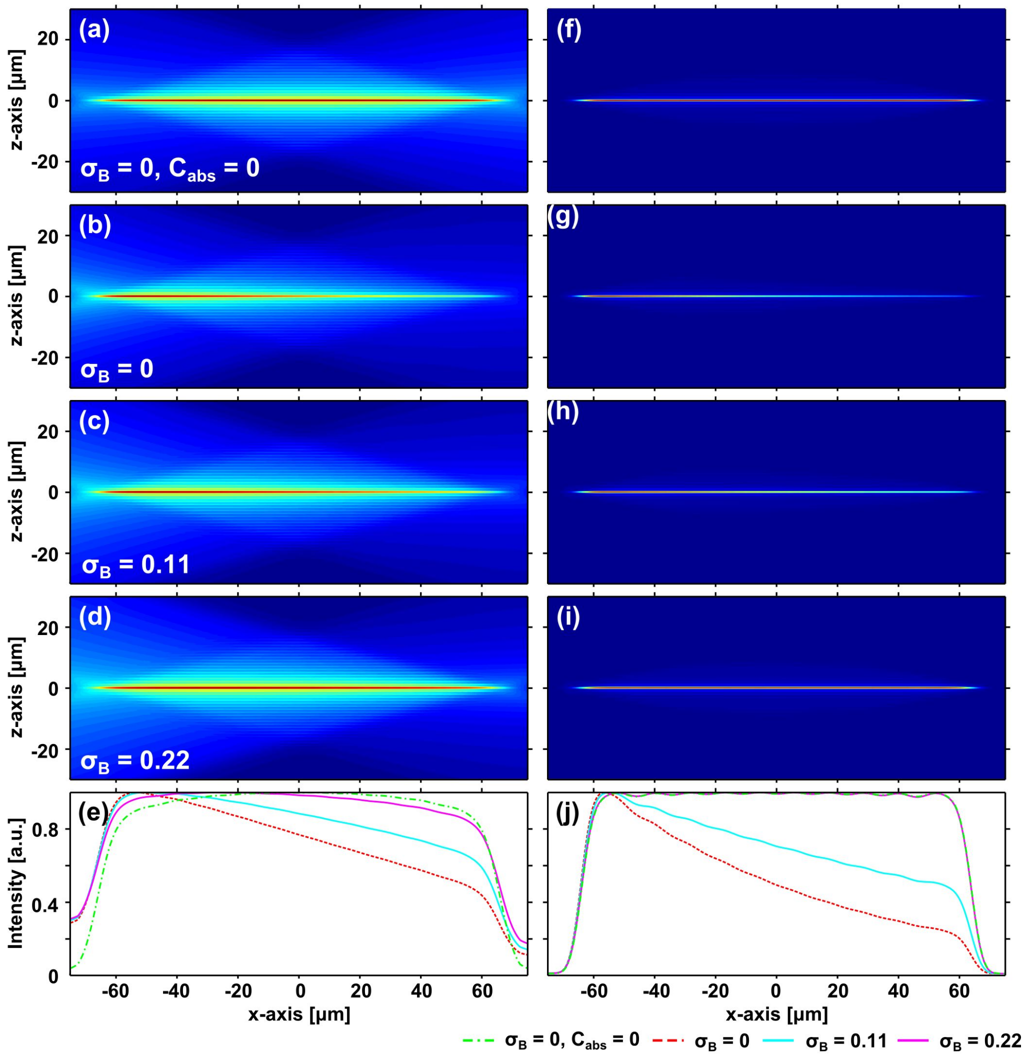

fig. S12. Illumination profiles (a-d) of attenuation-compensated 2PE Bessel light-sheets and their corresponding 2PE cross-sections, formed by digital scanning of the beams shown in Supp. Fig. S11, for no attenuation and no compensation (top row), attenuation ($C_{abs} = 65\text{cm}^{-1}$) and no compensation (2nd row), partial compensation (3rd row), and full compensation (4th row). On-axis intensity profiles for the illumination, and 2PE cross sections are shown in (e,j) respectively.

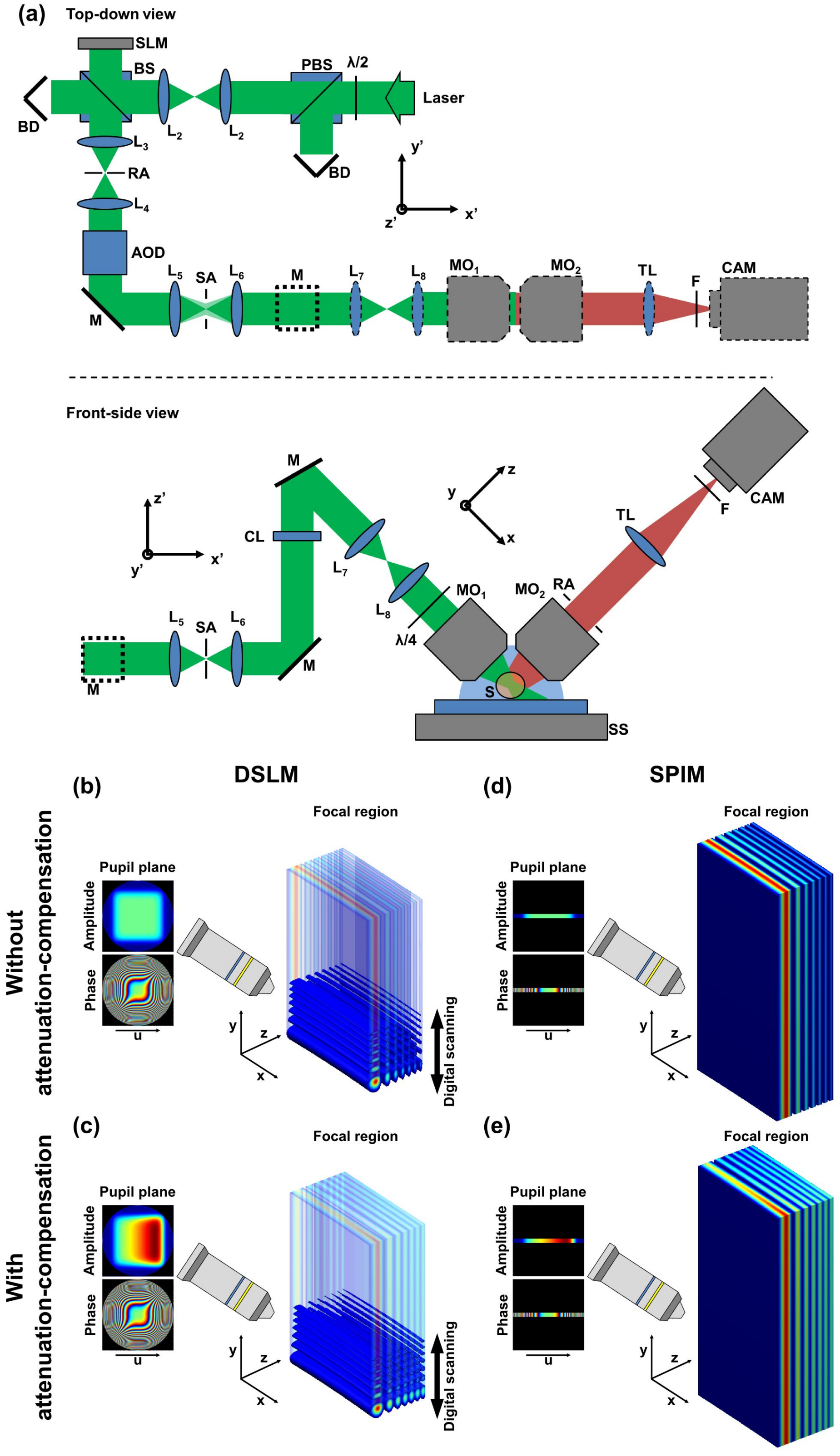

fig. S13. Schematic of attenuation-compensated Airy light-sheet microscope. (a) Top-down and front-side views of the optical setup (see Materials and Methods for (continued on next page)

Figure S13: (continued from previous page) full description). For DSLM operation, the cylindrical lens (CL) is removed and the light-sheet formation is as shown in (b, c) where a focused 2+1D Airy beam is rapidly scanned along the y-axis by the acousto-optic deflector (AOD). For SPIM operation, the cylindrical lens (CL) is reinserted which focuses the beam into a line on the final mirror perpendicular to the y-axis, the acousto-optic deflector (AOD) is switched off and the light-sheet is generated directly as shown in (d, e). AOD: acousto-optic deflector; BD: beam dump; BS: beam splitter (50:50); CAM: sCMOS camera; CL: cylindrical lens; F: fluorescence filter; L_x: lens; M: mirror; MO_x: microscope objective; PBS: polarizing beam splitter; RA: round aperture; S: sample; SA: slit aperture; SLM: spatial light modulator; SS: sample stage; TL: tube lens; $\lambda/2$: half-wave plate; $\lambda/4$: quarter-wave plate.

Supplementary Tables

Table #	Description	Page #
S1	Experimental parameters for all data shown in Main Text.	S30
S2	Field-of-view of 2-photon excitation SPIM and DLSM Airy light-sheet with attenuation and attenuation-compensation.	S31
S3	Field-of-view of 2-photon excitation Bessel beam and DLSM Bessel light-sheet with attenuation and attenuation-compensation.	S31

Fig. #	Sample	System type	Light-sheet parameters			Illumination power			Acquisition parameters		C_{attn}^{D} [cm^{-1}]
			α	σ	A_σ	P_{rel}^{Th}	P_{rel}^{Exp}	P [µW]	t_{int} [ms]	$z-$spacing [µm]	
3	Absorbing phantom	DLSM	7	0	0.48	1	1	140 ± 5	25	0.2	55
				0.23	0.70	1.38	1.37 ± 0.04	190 ± 5			
				0.46	1.07	2.21	2.26 ± 0.04	315 ± 5			
4	*S. lamarki*	DLSM	7	0	0.48	1	1	140 ± 5	50	0.4	85
				0.23	0.70	1.38	1.37 ± 0.04	190 ± 5			
				0.46	1.07	2.21	2.26 ± 0.04	315 ± 5			
5	*S. lamarki*	DLSM	7	0	0.48	1	1	140 ± 5	50	0.4	75
				0	0.71	2.19	2.14 ± 0.04	300 ± 5			
				0.46	1.07	2.21	2.26 ± 0.04	315 ± 5			
6	Mouse brain section	SPIM	7	0	0.48	1	1	1420 ± 10	100	0.2	100
				0.54	1.23	2.59	2.57 ± 0.02	3650 ± 10			

table S1. Experimental parameters for all data shown in Main Text. P_{rel}^{Th}: Relative power (with respect to a non-compensated Airy light-sheet ($\alpha = 7$, $\sigma = 0$, $A_\sigma = 0.48$)) determined by theory, P_{rel}^{Exp}: relative power determined experimentally, P: absolute experimental power, t_{int}: camera integration time, c_{attn}^{D}: estimated attenuation coefficient used in deconvolution procedure.

C_{abs}[cm^{-1}]	σ	FOV [µm] (1+1D Airy-SPIM)	FOV [µm] (2+1D Airy-DSLM)
0	0	230	192
65	0	98 (43%)	103 (54%)
65	0.285	150 (65%)	155 (81%)
65	0.57	230 (100%)	192 (100%)

table S2. Airy 2PE FOV given by the longitudinal FWHM for SPIM- and DSLM-style systems with various degrees of attenuation and attenuation-compensation (Supp. Fig. S10). The percentage in brackets is the FOV relative to the case of no attenuation.

C_{abs}[cm^{-1}]	σ_B	FOV [µm] (Bessel beam)	FOV [µm] (Bessel-sheet)
0	0	127	127
65	0	62.5 (49%)	62.5 (49%)
65	0.285	117 (92%)	117 (92%)
65	0.57	127 (100%)	127 (100%)

table S3. Bessel 2PE FOV given by longitudinal FWHM for Bessel beams (fig. S11) and Bessel-sheets (fig. S12) with various degrees of attenuation and attenuation-compensation. The percentage in brackets is the FOV relative to the case of no attenuation.
